# Information System for Symptom Diagnosis and Improvement of Attention Deficit Hyperactivity Disorder: Protocol for a Nonrandomized Controlled Pilot Study

**DOI:** 10.2196/40189

**Published:** 2022-09-28

**Authors:** Niki Pandria, Vasileia Petronikolou, Aristotelis Lazaridis, Christos Karapiperis, Eleftherios Kouloumpris, Dimitris Spachos, Anestis Fachantidis, Dimitris Vasiliou, Ioannis Vlahavas, Panagiotis Bamidis

**Affiliations:** 1 Medical Physics and Digital Innovation Lab, School of Medicine Faculty of Health Sciences Aristotle University of Thessaloniki Thessaloniki Greece; 2 Intelligent Systems Lab Department of Informatics Aristotle University of Thessaloniki Thessaloniki Greece; 3 The Second Method SA Thessaloniki Greece

**Keywords:** attention deficit hyperactivity disorder (ADHD), machine learning, web health, serious games, ADHD monitoring

## Abstract

**Background:**

Attention deficit hyperactivity disorder (ADHD) is one of the most common neurodevelopmental disorders during childhood; however, the diagnosis procedure remains challenging, as it is nonstandardized, multiparametric, and highly dependent on subjective evaluation of the perceived behavior.

**Objective:**

To address the challenges of existing procedures for ADHD diagnosis, the ADHD360 project aims to develop a platform for (1) early detection of ADHD by assessing the user’s likelihood of having ADHD characteristics and (2) providing complementary training for ADHD management.

**Methods:**

A 2-phase nonrandomized controlled pilot study was designed to evaluate the ADHD360 platform, including ADHD and non-ADHD participants aged 7 to 16 years. At the first stage, an initial neuropsychological evaluation along with an interaction with the serious game developed (“Pizza on Time”) for approximately 30-45 minutes is performed. Subsequently, a 2-week behavior monitoring of the participants through the mADHD360 app is planned after a telephone conversation between the participants’ parents and the psychologist, where the existence of any behaviors characteristic of ADHD that affect daily functioning is assessed. Once behavior monitoring is complete, the research team invites the participants to the second stage, where they play the game for a mean duration of 10 weeks (2 times per week). Once the serious game is finished, a second round of behavior monitoring is performed following the same procedures as the initial one. During the study, gameplay data were collected and preprocessed. The protocol of the pilot trials was initially designed for in-person participation, but after the COVID-19 outbreak, it was adjusted for remote participation. State-of-the-art machine learning (ML) algorithms were used to analyze labeled gameplay data aiming to detect discriminative gameplay patterns among the 2 groups (ADHD and non-ADHD) and estimate a player’s likelihood of having ADHD characteristics. A schema including a train-test splitting with a 75:25 split ratio, k-fold cross-validation with k=3, an ML pipeline, and data evaluation were designed.

**Results:**

A total of 43 participants were recruited for this study, where 18 were diagnosed with ADHD and the remaining 25 were controls. Initial neuropsychological assessment confirmed that the participants in the ADHD group showed a deviation from the participants without ADHD characteristics. A preliminary analysis of collected data consisting of 30 gameplay sessions showed that the trained ML models achieve high performance (ie, accuracy up to 0.85) in correctly predicting the users’ labels (ADHD or non-ADHD) from their gameplay session on the ADHD360 platform.

**Conclusions:**

ADHD360 is characterized by its notable capacity to discriminate player gameplay behavior as either ADHD or non-ADHD. Therefore, the ADHD360 platform could be a valuable complementary tool for early ADHD detection.

**Trial Registration:**

ClinicalTrials.gov NCT04362982; https://clinicaltrials.gov/ct2/show/NCT04362982

**International Registered Report Identifier (IRRID):**

RR1-10.2196/40189

## Introduction

### Background and Objectives

Attention deficit hyperactivity disorder (ADHD) is one of the most common neurodevelopmental disorders during childhood [1]. ADHD is commonly diagnosed during childhood and prolongs into adulthood to a variable extent ranging from 5% to 75% [2]. ADHD is characterized by persistent symptoms of inattention and hyperactivity/impulsivity that interfere with or attenuate social, academic, and occupational functioning, as well as the developmental stage. The symptoms are present prior to the age of 12 years, for a period of at least 6 months in 2 or more settings [3].

The diagnosis procedure remains challenging, as it is nonstandardized, multiparametric, and highly dependent on subjective evaluation of the perceived behavior [4,5]. Additionally, the effectiveness of treatment strategies commonly relies on systematic monitoring using pen-and-paper methods [4,6]. Subjectivity bias, difficulty monitoring in different settings, and risk of data loss are inherent obstacles to these methods.

To address the limitations of the existing approaches, ADHD360 focuses on developing an integrated technology solution that includes a serious game for a probabilistic prediction indicating the presence of ADHD in players using machine learning (ML) models and a mobile app for monitoring ADHD behaviors. The platform aims to (1) facilitate early detection of ADHD characteristics and (2) serve as an adjunct intervention for ADHD management. The first goal of the project is to analyze the gameplay behavior of the users with respect to their diagnosis (ie, ADHD or non-ADHD). Thus, discriminative gameplay patterns among the groups (ADHD or non-ADHD) are explored to estimate the users’ likelihood of having ADHD characteristics. The second goal is to investigate the effectiveness of the platform as an intervention for ADHD management.

### Literature Summary

Discrepancies regarding ADHD prevalence have emerged over time and among studies, leading to mixed conclusions about the possible underdiagnosis or overdiagnosis of the disorder [7]. Multiple factors have been identified to affect the recognition and diagnosis of ADHD including the parental role, school-based factors, intrinsic factors related to children, and the role of health providers [8].

In terms of the medical aspects, the access to health providers [7], limited reimbursement for specialized mental care [9], differences in clinical approaches, scoring cutoff, and factors related to the medical systems across different countries [10] have contributed to the existing difficulties in the diagnosis process. However, the underlying inconsistencies in the definition of ADHD based on different diagnostic manuals make the recognition and diagnosis of ADHD even more challenging [3,11]. Moreover, ADHD diagnosis could be biased due to the underlying subjectivity of the assessment and information interpretation procedures [12], and the lack of standardization [11].

Although diagnostic challenges could be addressed through a detailed history of prenatal conditions, family status and school/academic life [7,13], standardized tools that could enhance the diagnosis accuracy are necessary.

As early detection of ADHD could ameliorate the disorder’s development, diminish its long-term impact [14], improve quality of life [15], and enhance overall functioning and self-esteem [16,17], research efforts were focused on formulating programs [14,18,19], games [20,21], or game-based tools [22,23] for early diagnosis.

For addressing the existing challenges, ADHD360 aims to develop an integrated platform comprising a serious game and a mobile app for monitoring ADHD behaviors in a SMART (Specific, Measurable, Attainable, Realistic, and Timely) way [24,25] as the core elements. The design of the serious game is based on the Diagnostic and Statistical Manual of Mental Disorders, Fifth Edition (DSM-V) [3] as well as neuropsychological tools. The basic principles of DSM-V and common neuropsychological tools can be easily transferred to the game design process via game mechanics implementation focusing on a specific ADHD behavior.

## Methods

### Study Design

A 2-phase nonrandomized controlled pilot study was performed in the context of the ADHD360 project. The protocol of the pilot trials was initially designed for in-person participation, but after the COVID-19 outbreak, it was adjusted to the new conditions imposed by the pandemic, providing remote participation. The procedures for each of these 2 ways of participation are described thoroughly below.

#### In-Person Participation

The first part of the pilot study begins with a first meeting between the involved members of the research team and the parents of each participant at the Laboratory of Medical Physics and Digital Innovation, School of Medicine of Aristotle University of Thessaloniki (iMedPhys). The aim of this first meeting is to thoroughly inform the parents and the children regarding the nature and scope of the project, as well as the exact procedures that take place according to the research protocol. In this context, parents can ask questions, express any concerns regarding the project, and sign the informed consent form. Afterward, the participants are subjected to a brief neuropsychological assessment by an experienced psychologist from the research team. This neuropsychological assessment includes 6 subtests of the Wechsler Intelligence Scale for Children, Fifth Edition (WISC-V), an intelligence test that measures a child’s intellectual ability, and 5 cognitive domains that impact performance [26]. The 6 subtests of WISC-V used here are (1) similarities, (2) vocabulary, (3) block design, (4) figure weights, (5) digit span, and (6) coding. The main aim of this assessment is to obtain a more integrated view of the participants’ general intellectual abilities in several domains such as verbal comprehension, visual-spatial perception, working memory, processing speed, and fluid reasoning. The average time to complete the subtests is approximately 48-50 minutes. If any of the participants have been assessed by this scale during the past 2 years, they are not reassessed by our research team, and the children’s parents are kindly asked to provide the already existing scores of the aforementioned 6 subtests, if possible. Furthermore, the ADHD Rating Scale-IV [27] is a 4-point Likert brief questionnaire completed by the parents regarding the presence and frequency of ADHD symptoms. Inattention and hyperactivity-impulsivity are the 2 subscales integrated into the ADHD Rating Scale-IV. The total raw score of the scale is calculated by summing the scores of the 2 subscales.

Subsequently, the participants interact with the serious game “Pizza on Time” that has been specifically developed for the ADHD360 project for approximately 30-45 minutes at iMedPhys. During this interaction with the serious game, a form is completed regarding the conditions, the duration and the behaviors that will possibly be occurred. Participants interact again with the serious game for 30-45 minutes at iMedPhys, continuing from the level at which they stopped at the previous visit to complete all the levels of the first setting (the urban setting).

After the aforementioned procedures, there is a telephone conversation between the participants’ parents and the psychologist who conducted the neuropsychological evaluation to discuss if there are any behaviors characteristic of ADHD that affect the daily functioning of the participants. Once the psychologist has reached a consensus with the parents that there are certain behaviors that could be observed, the behaviors are introduced into the participant's account in the mADHD360 app. Instructions regarding the installation and use of the mobile app are provided by the research team through email. The mADHD360 app can be used on a smartphone or tablet. An observation plan is scheduled, including at least 2-4 times per week of behavioral monitoring regarding the frequency or the duration of these behaviors during the time they usually spend with the participant. Each round of behavior monitoring has a duration of 12 minutes. The duration of this phase is determined to be 2 weeks. During the behavior monitoring phase, the participants do not interact with the serious game.

Once behavior monitoring is complete, the research team instructs the participants to continue playing the game using the second (jungle) and third (space) settings on the premises of iMedPhys. The mean duration of this part is 10 weeks with a frequency of at least 2 times a week, depending on the participants’ availability. In this second part of the pilot testing phase, the goal is to exploit the serious game as an intervention to improve certain ADHD symptoms.

Next, a second round of behavior monitoring is performed following the same procedures as the previous one. Furthermore, a neuropsychological assessment is conducted following the same procedures as those during the first evaluation in the beginning of their participation.

#### Remote Participation

All the procedures are identical to those followed during in-person participation, apart from the interactions with the serious game that are done remotely from their home using their personal computers.

### Participant Selection

The eligibility criteria were reviewed by a member of the research team before assignment to the study. Textbox 1 lists the inclusion and exclusion criteria for enrollment into the study.

**Inclusion and exclusion criteria for participant enrollment.**

**Inclusion criteria**

- Participants should be between 7 and 16 years old.
- Participants of the attention deficit hyperactivity disorder (ADHD) group should be diagnosed by an approved body of the Ministry of Health.
- Participants should be willing to follow the study protocol and procedures.
- The ADHD symptoms should not be attributed to organic disease.
- Parents must voluntarily provide written consent for their child’s participation in the study.

**Exclusion criteria**

- Participants suffering from comorbid conditions other than ADHD are excluded.
- Children whose parents are not willing to provide written consent for participation in the study are excluded.

All participants are comprehensively informed regarding the procedures of the study through a document named “Participant Information and Consent Form.” Moreover, the participants are informed about the purpose of the study as well as the following aspects of their participation:

- Their participation is voluntary.
- They can ask questions about the study procedures before participating in the study.
- They will be aware of the underlying risk or burden associated with their participation.
- They will know who will benefit from conducting this research.
- They will know how data collection and data protection will be performed during the project’s lifetime as well as whether data will be destroyed or reused (in case there is a possibility to reuse them).
- They will be fully informed, and they will agree for further use of their data.
- They can leave the study at any time and withdraw their data from the study.
- They will be aware of the possible commercial exploitation of the research findings.

Each informed consent is signed by the researcher who informs the participant and the principal investigator of the study. The informed consent explicitly states that the study was approved by the Committee for Bioethics and Ethics of the Medical School at the Aristotle University of Thessaloniki and all personal details are anonymized using a unique user identification code.

### Ethics Approval

The study protocol (trial registration: NCT04362982) was approved by the Ethics and Bioethics Committee of the School of Medicine at the Aristotle University of Thessaloniki (reference number: 6.225/29.7.2020).

### Recruitment of Participants

We initially considered recruiting at least 20 participants (10 ADHD and 10 non-ADHD) in our pilot trials. We estimated the sample size for the definite study by performing power analysis using GPower software (version 3.1, Universität Düsseldorf). We conducted a paired *t* test analysis using a power of 0.8, a significance level of 0.05, and an effect size equal to 0.2. The total sample size was estimated to be 199 participants. As proposed by Billingham et al [28], the sample size in pilot trials does not need calculation but needs justification. Thus, recruiting a small number of participants seems sufficient according to the suggestions of Stallard [29] and is similar to what was proposed by Julious [30].

The ADHD group is mainly composed of patients from the Community Center for Mental Health of Children and Adolescents at the General Hospital of Thessaloniki “G. Papanikolaou,” which supports the implementation of pilot trials. Furthermore, dissemination activities such as social media posts, mass media interviews, information events open to the public, and project presentations at conferences and workshops are considered essential to recruit participants for pilot trials.

### ADHD360 Platform Design

The ADHD360 platform consists of 2 main components, a serious game and a mobile app for behavior monitoring.

#### Serious Game for ADHD

Game design covers a wide range of activities in designing games, including story, aesthetics, mechanics, and technology [31]. These variables have to be carefully considered for successful game design. Hunicke et al [32] propose the mechanics-dynamics-aesthetics framework and define game mechanics as the “mechanisms that describe the specific elements of the game, at the level of data presentation and algorithms (...), also the mechanics relate to the behaviors and control mechanisms provided to the player in the game context.”

The mechanics-dynamics-aesthetics framework standardizes the basic components of a game into distinct parts such as rules, systems, and fun. This distinction helps us in the design of the mechanisms because the basic components of the game can be translated into game design elements such as mechanics, dynamics, and aesthetics that are visible to both the end users and the game designer, but from different perspectives. There are some games that have been developed specifically to improve ADHD symptoms, whereas commercial game titles have also been used for research purposes to measure the performance of users with or without ADHD diagnosis [33].

The 2 games that focus on improving ADHD symptoms are Plan it Commander [34] and Antonyms [35]. The 2 major gaps in existing research are the (1) interconnection of the game mechanics with the diagnostic criteria according to DSM-V and (2) correlation of user performance with the standard results of ADHD tests through ML algorithms.

**Figure 1:**
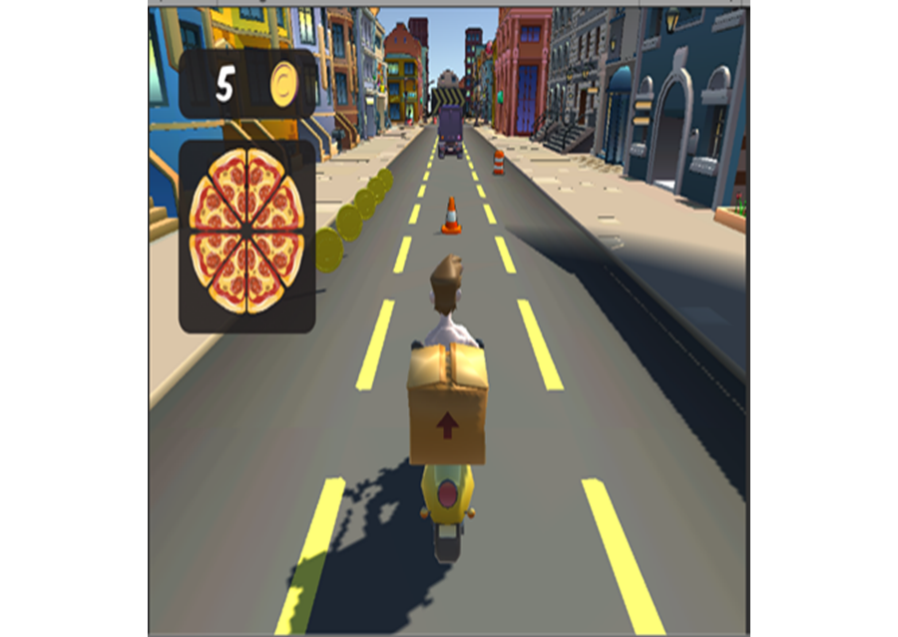
Screenshot of the “Pizza on Time” game.

For bridging this gap, we focused on developing a serious game called “Pizza on Time” (Figure 1), which is a runner game. The player tries to avoid obstacles and collect coins to deliver the pizza, and each time the player hits an obstacle, a pizza slice is lost. When all the pizza slices are lost, the player needs to start over at the same level. There are 3 different in-game world levels (city, jungle, and space) with a total of 120 predefined sublevels. Additionally, 4 mini games were integrated with the main runner game to enrich the existing mechanics, retaining the concept of pizza delivery.

#### Mobile App for Behavior Monitoring

The ADHD360 monitoring app (mADHD360) developed in the project provides teachers, parents, and health professionals with features to easily monitor specific targeted behaviors related to ADHD. Although several related information and communications technology solutions were implemented to replace traditional pen-and-paper observation charts in the past years [24], not all of them were successful [6]. The mADHD360 app differs from other digital monitoring apps, which are mainly based on pre-existing traditional forms of assessment. Instead, the app addresses the need to include the range of observation features needed to conduct a complete functional behavioral assessment in a unique and an easy-to-use tool. Finally, it allows teachers, parents, and clinicians to use a sociocentric technology to create a network of people and develop and monitor a behavioral intervention plan that is shared among all people involved in the care of the child with ADHD.

The process of creating a new subject profile and monitoring behavior can be described as follows:

First, the associate user (usually one of the parents) creates an account and enters the basic data of the subject to be monitored in the mADHD360 app. There is a strict policy regarding the sensitive personal data of the children; it does not allow real names to be used or any other significant detail that can reveal the identity of the children in the real world.

The next step is to create a network of people, usually people that spend time with the child (such as a relative or a class teacher) and a health professional. The app supports a directory of ADHD experts, which can be added to the child’s observation network. Inviting other people is a very simple process, and it can be performed via email.

A limited number of ADHD-related behaviors are associated with the child. The associations come from a predefined vocabulary [36], but the user can also enter custom behaviors. The network of people can gather data to unveil the function of a child‘s behavior and plan an intervention to reduce or eliminate the undesirable behavior with the help of a health professional. Data are gathered in sessions, and for each behavior, the app can monitor the frequency and duration. Usually, there are 2 rounds of monitoring, 1 before the intervention and the other during or even after the intervention.

Finally, the efficacy of each treatment can be evaluated by health professionals through visual analysis of the data gathered by the network members during the assessment periods.

The mADHD360 app is based on the Web Health Application for ADHD Monitoring (WHAAM) [36]; although the basic principles are retained, the latter is enhanced with more features, such as asynchronous chat capabilities, and offers advanced usability and user experience.

From a technology point of view, the mADHD360 app is designed to run on mobile devices, smartphones, and tablets. It is delivered for free as a progressive web app with the main game.

### Analysis

#### Statistical Analysis

Statistical analysis is performed using SPSS (version 26.0, IBM Corp) and the statistical significance level is set at *P*=.05. Continuous variables are explored for normality by the Shapiro-Wilk test to calculate the appropriate descriptive statistics. Continuous variables that are approximately normally distributed are reported as means (SDs) whereas those that are not normally distributed as medians and IQRs (Q1-Q3, where Q1 and Q3 are the first and the third distribution quartiles, respectively). Neuropsychological data expressed as raw scores are treated as continuous variables. Categorical data are described as frequencies and percentages.

#### ML Methodology

State-of-the-art ML algorithms are used to analyze labeled gameplay data. Specifically, we attempt to classify game-generated data based on the user’s known label (ADHD or non-ADHD). All in-game events of a gameplay session are recorded to form a time series that one can use to reconstruct the whole gameplay session. These events correspond to player actions and environmental parameters, such as performing a jump/move, left or right moving action, obstacle collision, and coin collection. Informative feature extraction is performed by splitting players’ time series depending on the game level. Different feature extraction techniques are assumed for each level depending on its type. In the main runner game, several aggregates are calculated by capturing different events during the gameplay (ie, movement of a player or the ability to avoid obstacles). In the mini games, feature extraction is based on the characteristic game mechanics and events of the respective mini game. The final feature vector of each player is produced by concatenating the individual-level feature vectors.

A schema that includes train-test splitting with a 75:25 split ratio, *k*-fold cross-validation (CV) with k=3, an ML pipeline, and data evaluation was designed for experimental purposes. The train-test splitting is used for model specification (eg, feature selection and model tuning), model selection, and training of the final model. Modeling decisions are made on the training set. Thus, the test set is used only to evaluate and report the statistics of the algorithm’s performance. The purpose of 3-fold CV is to fine-tune the hyperparameters of the supervised learning algorithms (classifiers) that we investigate.

For each classifier, an ML pipeline was designed to include the following procedures: (1) removal of features with 0 variance, (2) univariate feature selection according to a statistical criterion (ANOVA *F* value between label and feature), (3) principal component analysis for dimensionality reduction, and (4) classifier training. Therefore, the hyperparameters of all the steps are tuned (eg, the number of components in principal component analysis) in the 3-fold CV process. The *F1* score is used as the optimization criterion in the 3-fold CV, and the best configuration of each method's pipeline is then used on the entire training set (fully trained pipeline).

Next, the algorithm with the best-performing pipeline in the 3-fold CV is selected as the proposed method. The general performance of our system on unlabeled data is estimated by evaluating the predictive performance of the proposed method that consists of a fully trained pipeline on the test set. The performance of the ML system is also evaluated based on methods other than the proposed one. The following learning algorithms were applied: k-nearest neighbors (kNN) [37], logistic regression (LogReg) [38], support vector machine (SVM) [39], random forest (RF) [40], ridge classifier (RC) [41], passive aggressive classifier (PAC) [42], stochastic gradient descent (SGD) [43], and naïve Bayes (NB) [44]. For each classifier, we tuned its most important parameters using grid-search CV on a sufficient range of parameter values. The objective of the optimization is to maximize the micro-*F1* score across all validation folds. In addition, we report the performance metrics of accuracy, precision, recall, and *F1* scores.

## Results

### Demographics

In total, 43 participants were recruited with a mean age of 11.82 (SD 2.81) years. Among these, 28 (65%) were male and 15 (35%) were female. Of the 43 participants, 18 (42%) were diagnosed with ADHD and 25 (58%) participants were considered controls (non-ADHD). Approximately 30 participants (70%) completed the first part of the pilot phase remotely.

### Neuropsychological Data

Neuropsychological data were obtained from 30 participants. They were evaluated using WISC-V by the psychologist of the ADHD360 team or by an approved national body within the last 2 years. Among the 30 participants, 10 (33%) were diagnosed with ADHD and 20 (67%) were considered as healthy controls (non-ADHD). There were 21 males (70%) and 9 females (30%). In the ADHD group, all participants were male, whereas in the non-ADHD group, 11 (55%) were male and 9 (45%) were female. The mean age of the screened participants was 11.40 (SD 2.85) years. In the ADHD group, the mean age of the participants was 12.90 (SD 2.64) years, whereas that of the participants in the non-ADHD group was 10.65 (SD 2.70) years.

According to the data from the first neuropsychological assessment, the mean score in the figure weights subtest in the ADHD group was 14.00 (SD 5.79), whereas the median score of this subtest in the non-ADHD group was 19 (IQR 13-24). The mean scores in the subtests block design, similarities, digit span, coding, and vocabulary are presented in Table 1.

The mean score of the participants in the inattention subscale of the ADHD Rating Scale-IV was 12.70 (SD 7.04), whereas that in the hyperactivity-impulsivity subscale was 10.97 (SD 6.65). In the full group of participants, the mean total score was 23.67 (SD 12.56). The mean scores on the ADHD Rating Scale-IV obtained by grouping the participants based on their diagnosis are presented in Table 2.

**Table 1:** Mean scores of the study participants in the subtests block design, similarities, digit span, coding, and vocabulary.

WISC^a^ subtest	ADHD^b^, mean (SD)	non-ADHD, mean (SD)
Block design	19.60 (4.30)	22.85 (8.02)
Similarities	22.00 (7.45)	23.50 (9.05)
Digit Span	18.30 (4.03)	19.75 (6.26)
Coding	27.80 (9.69)	32.20 (13.46)
Vocabulary	20.20 (5.55)	23.05 (8.17)

^a^WISC: Wechsler Intelligence Scale for Children.

^b^ADHD: attention deficit hyperactivity disorder.

**Table 2:** Mean scores of the participants on the ADHD Rating Scale-IV.

ADHD^a^ Rating Scale-IV	ADHD, mean (SD)	non-ADHD, mean (SD)
Inattention subscale	18.50 (5.84)	9.80 (5.74)
Hyperactivity-impulsivity subscale	15.30 (6.58)	8.80 (5.67)
Total score	33.80 (9.94)	18.60 (10.62)

^a^ADHD: attention deficit hyperactivity disorder.

### Gameplay Data

Following the methodology described above, we present the results of a preliminary analysis of gameplay data collected during pilot trials. Gameplay data were collected from all recruited participants. However, technical difficulties (ie, unstable internet connection and game installation issues) led to insufficient or no data generation in 13 of the 43 participants (30%). Therefore, gameplay data collected from 30 (70%) participants were used in the following preliminary analysis. We comprehensively describe the CV results for a training set consisting of data collected from 22 of the 30 participants (73%) as well as the selection of the best model. Subsequently, we evaluate the predictive performance on a test set consisting of data gathered from 8 (27%) participants using point estimates and CIs.

**Figure 2:**
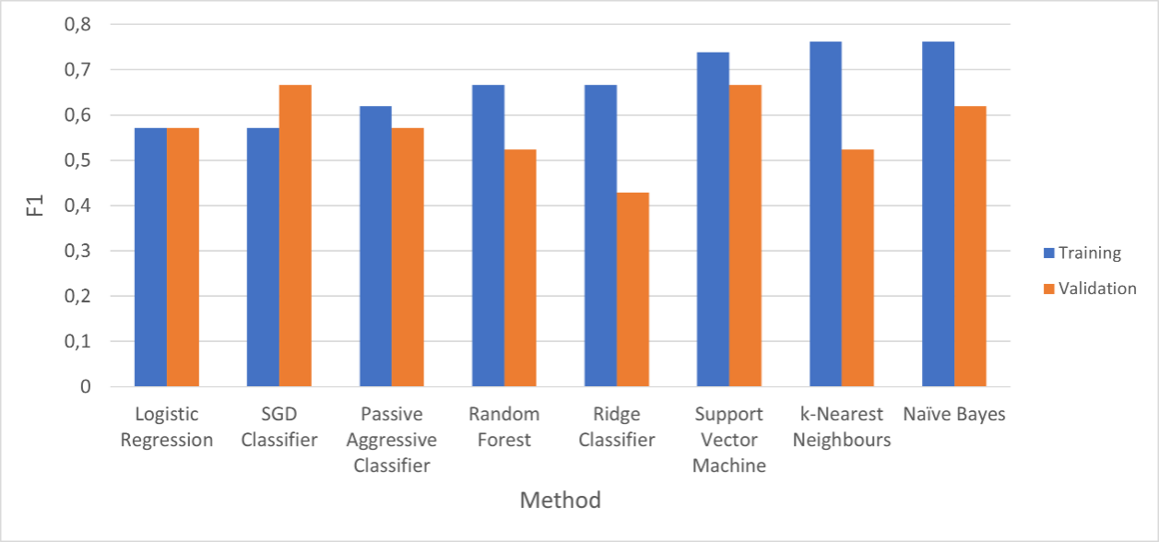
Cross-Validation F1 scores. SGD: stochastic gradient descent.

A graphical illustration of the CV *F1* results for each learning method is shown in Figure 2. Furthermore, detailed results of the *F1* score and the other performance measures for the CV procedure are given in Table 3.

Most classifiers achieved an *F1* score above 0.5. We observed significant overfitting for RF, kNN, and NB, with a difference of 0.1 between the training and validation scores. The test performance scores are given in Table 4.

The previously mentioned models seem to be substandard solutions for this classification problem, with all of them dominated by LogReg, SGD, SVM, and PAC. The latter models feature a tradeoff between validation performance and goodness of fit. Among these methods, we selected SVM for the following reasons: (1) It achieves the highest validation *F1* score (0.66). (2) Although SGD achieves the same validation score as SVM, the latter also achieves a better fit with a difference of approximately +0.07 between training and validation, which is approximately –0.09 for the former.

Even though the results are indicative of the potential that ML has in the domain of ADHD prediction based on gameplay data, we presume that experimenting with a larger data set could give more accurate and concrete conclusions.

**Table 3:** Cross-validation results.

Method	Accuracy		Precision			Recall			*F1*	
	Training	Validation	Training	Validation	Training		Validation	Training		Validation
NB^a^	0.7619	0.6190	0.7011	0.5556	0.8333		0.7778	0.7619		0.6190
k-NN^b^	0.7619	0.5238	0.7083	0.4500	0.7778		0.5556	0.7619		0.5238
LogReg^c^	0.5714	0.5714	0.0000	0.0000	0.0000		0.0000	0.5714		0.5714
PAC^d^	0.6190	0.5714	0.5337	0.5000	0.8333		0.8889	0.6190		0.5714
RF^e^	0.6667	0.5238	0.5935	0.4444	0.7222		0.5556	0.6667		0.5238
RC^f^	0.6667	0.4286	0.6074	0.3833	0.6667		0.5556	0.6667		0.4286
SGD^g^	0.5714	0.6667	0.4932	0.5667	0.6667		1.0000	0.5714		0.6667
SVM^h^	0.7381	0.6667	0.7238	0.5833	0.6667		0.6667	0.7381		0.6667

^a^NB: naïve Bayes.

^b^k-NN: k-nearest neighbors.

^c^LogReg: logistic regression.

^d^PAC: passive aggressive classifier.

^e^RF: random forest.

^f^RC: ridge classifier.

^g^SGD: stochastic gradient descent.

^h^SVM: support vector machine.

**Table 4:** Evaluation of test results.

Method	Accuracy		Precision			Recall			*F1*	
	CI	Test	CI	Test	CI		Test	CI		Test
NB^a^	0.14-0.85	0.42	0.14-0.85	0.42	1.00-1.00		1.00	0.25-0.92		0.60
k-NN^b^	0.14-0.71	0.42	0.00-1.00	0.33	0.00-1.00		0.33	0.00-0.67		0.33
LogReg^c^	0.14-0.85	0.42	0.14-0.85	0.42	1.00-1.00		1.00	0.25-0.92		0.60
PAC^d^	0.13-0.71	0.28	0.13-0.75	0.33	0.23-1.00		0.66	0.23-0.83		0.44
RF^e^	0.28-0.85	0.57	0.15-1.00	0.50	0.23-1.00		0.66	0.23-0.90		0.57
RC^f^	0.27-0.85	0.57	0.00-1.00	0.50	0.00-1.00		0.66	0.00-0.88		0.57
SGD^g^	0.14-0.71	0.42	0.00-1.00	0.33	0.00-0.76		0.33	0.00-0.66		0.33
SVM^h^	0.71-1.00	0.85	0.50-1.00	0.75	1.00-1.00		1.00	0.66-1.00		0.85

^a^NB: naïve Bayes.

^b^k-NN: k-nearest neighbors.

^c^LogReg: logistic regression.

^d^PAC: passive aggressive classifier.

^e^RF: random forest.

^f^RC: ridge classifier.

^g^SGD: stochastic gradient descent.

^h^SVM: support vector machine.

## Discussion

Limitations of the existing screening procedures for ADHD diagnosis are summarized as high dependence on pen-and-paper practices, subjectivity bias, difficulty in monitoring in different settings, and risk of data loss. The ADHD360 platform, consisting of the serious game “Pizza on Time” and the mADHD360 app, attempts to offer a holistic technological solution for early detection of ADHD characteristics and serve as a training tool against ADHD-related symptoms. It facilitates real-time data collection in different settings and from different individuals involved in the user’s care. Moreover, the ADHD360 platform enables quantitative data analysis assessing the behavior and game performance of the user while providing a more integrated view of the user's behavioral characteristics. Investigating different state-of-the-art ML methods revealed that our platform is characterized by a notable capacity to discriminate players based on their in-game patterns as those who have ADHD characteristics and those who do not. Thus, it is expected to serve as a complementary screening tool benefiting health care professionals, educators, and people with ADHD.

However, the health emergency imposed by COVID-19 affected the implementation of pilot trials by forcing adaptation of the protocol to remote conditions. Moreover, lockdowns and social distancing may have contributed to the low sample size and the high number of dropouts during the study. Additionally, the long duration of the experimental protocol and the need for commitment to the study procedures may have negatively affected users’ adherence to pilot trials. Moreover, ADHD has been characterized by “a dislike of mental effort” [45]. Considering the aforementioned limitations, we recruited about twice as many participants as originally intended. However, the dropout rate was approximately 30% (13 participants), as 30 participants (70%) completed the first part of pilot phase remotely.

Even though preliminary analyses of the collected data have shown promising results, the platform can be further improved by training the ML models on a larger data set that can be developed by recruiting more participants. This would allow the learning models to further improve their accuracy in correctly distinguishing ADHD gameplay behaviors from non-ADHD gameplay behaviors. The monitoring app will soon have a dedicated enrollment service for health experts; parents will be able to see a list of related professionals near their area and contact a person to join their child’s network. Moreover, we will add asynchronous chatting capabilities and a strict schedule-monitoring functionality, with device and calendar notifications. The “Pizza on Time” game is planned to be released to the public for both Android and iOS devices, as well as for PCs (Windows and Mac).

## Appendix

Multimedia Appendix 1 Peer-review report by the Special Management Service for the Competitiveness, Entrepreneurship and Innovation Operational Programme (Ειδική Υπηρεσία Διαχείρισης Επιχειρησιακού Προγράμματος Ανταγωνιστικότητα Επιχειρηματικότητα και Καινοτομία (ΕΥΔ ΕΠΑνΕΚ)) - Special Service for the Management of Implementation of Research, Technological Development and Innovation Activities (Ειδική Υπηρεσία Διαχείρισης και Εφαρμογής Δράσεων στους τομείς Έρευνας, Τεχνολογικής Ανάπτυξης και Καινοτομίας (ΕΥΔΕ ΕΤΑΚ)) (Greece).

## Abbreviations

ADHD: attention deficit hyperactivity disorder
CV: cross-validation
DSM-V: Diagnostic and Statistical Manual of Mental Disorders, Fifth Edition
iMedPhys: Medical Physics and Digital Innovation, School of Medicine of Aristotle University of Thessaloniki
kNN: k-nearest neighbors
LogReg: logistic regression
ML: machine learning
NB: naïve Bayes
PAC: passive aggressive classifier
RC: ridge classifier
RF: random forest
SGD: stochastic gradient descent
SVM: support vector machine
WISC-V: Wechsler Intelligence Scale for Children, Fifth Edition
