# Supplementary material for: Information System for Symptom Diagnosis and Improvement of Attention Deficit Hyperactivity Disorder: Protocol for a Nonrandomized Controlled Pilot Study
Source: JMIR Res Protoc. 2022 Sep 28;11(9):e40189. doi: 10.2196/40189 (PMC9557982; doi:10.2196/40189)
Supplement: Multimedia Appendix 1 [file resprot_v11i9e40189_app1.docx]

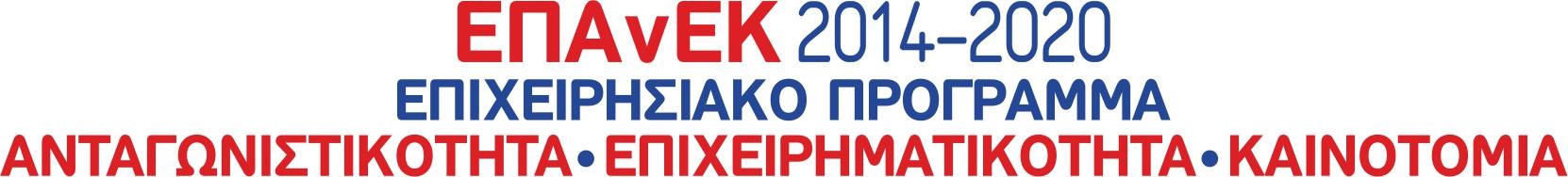


# ΔΡΑΣΗ ΕΘΝΙΚΗΣ ΕΜΒΕΛΕΙΑΣ:

**«ΕΡΕΥΝΩ-ΔΗΜΙΟΥΡΓΩ-ΚΑΙΝΟΤΟΜΩ»**

# Ειδική Υπηρεσία Διαχείρισης Επιχειρησιακού Προγράμματος Ανταγωνιστικότητα Επιχειρηματικότητα και Καινοτομία (ΕΥΔ ΕΠΑνΕΚ)

**Ειδική Υπηρεσία Διαχείρισης και Εφαρμογής Δράσεων στους τομείς Έρευνας, Τεχνολογικής Ανάπτυξης και Καινοτομίας**

# (ΕΥΔΕ ΕΤΑΚ)

**ΑΞΙΟΛΟΓΗΣΗ ΠΡΟΤΑΣΗΣ ΕΡΕΥΝΗΤΙΚΟΥ ΕΡΓΟΥ**

**ΤΙΤΛΟΣ: Ολοκληρωμένη πλατφόρμα διαγνωσης και βελτίωσης των συμπτωμάτων της Διαταραχής Ελλειμματικής Προσοχής και Υπερκινητικότητας**

**ΑΚΡΩΝΥΜΙΟ: ADHD360**

**ΚΩΔΙΚΟΣ ΕΡΓΟΥ Τ1ΕΔΚ-01680**


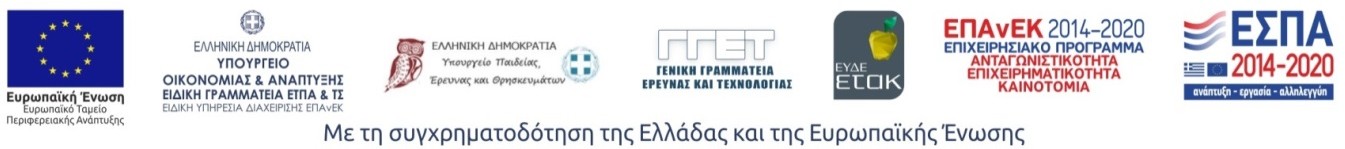


**ΑΚΡΩΝΥΜΙΟ ΠΡΟΤΑΣΗΣ** ADHD360

**Τίτλος (Ελληνικά)** Ολοκληρωμένη πλατφόρμα διαγνωσης και βελτίωσης των συμπτωμάτων της

Διαταραχής Ελλειμματικής Προσοχής και Υπερκινητικότητας

**Τίτλος (Αγγλικά)** Information System for symptom diagnosis and improvement of Attention Deficit

Hyperactivity Disorder

# ΠΕΡΙΛΗΨΗ ΠΡΟΤΑΣΗΣ

Η ΔΕΠΥ είναι μία από τις συχνότερες νοητικές διαταραχές και χαρακτηρίζεται κυρίως από τα συμπτώματα μειωμένης ικανότητας προσοχής. Μέχρι σήμερα, η διάγνωση, οι παρεμβάσεις και η αξιολόγηση της αποτελεσματικότητας αυτών, βασίζονται σε συστηματικές καταγραφές ειδικών. Οι συστηματικές καταγραφές, αν και προσφέρουν κρίσιμα στοιχεία στη διάγνωση και αντιμετώπιση της ΔΕΠΥ, εντούτοις συχνά εμπεριέχουν υποκειμενικότητα. Η υποκειμενικότητα προέρχεται από το γεγονός ότι οι συστηματικές καταγραφές βασίζονται κυρίως σε ποιοτικά χαρακτηριστικά, όπως είναι οι απαντήσεις ενός παιδιού που πάσχει, καθώς και των γονιών του, στα ερωτήματα του ειδικού.

Βασικό αντικείμενο του έργου είναι η ανάπτυξη της ολοκληρωμένης τεχνολογικής πλατφόρμας, με τίτλο ADHD360, με κεντρικό στοιχείο ένα παιχνίδι σοβαρού σκοπού (serious game). Η λειτουργικότητα που θα υλοποιηθεί στην ADHD360 αφορά α) τη διάγνωση της διαταραχής β) την αντιμετώπιση των συμπτωμάτων μέσω της ίδιας της

εφαρμογής αλλά και οργανωμένων παρεμβάσεων και γ) την αξιολόγηση της αποτελεσματικότητας αυτών.

Η αλληλεπίδραση των χρηστών με την εφαρμογή παιχνιδιού σοβαρού σκοπού θα παράγει εκείνα τα δεδομένα που θα συλλαμβάνουν τα μετρήσιμα χαρακτηριστικά της ΔΕΠΥ. Τα δεδομένα αυτά θα τροφοδοτήσουν μια σειρά από στατιστικές αναλύσεις για (α) την προσαρμογή των κανόνων του παιχνιδιού (game mechanics) και (β) την κατασκευή διαγνωστικών μοντέλων με την χρήση καινοτομικών μεθόδων Μηχανικής Μάθησης. Τα μοντέλα θα ενσωματωθούν στην ADHD360, ώστε τελικά να παρέχονται υπηρεσίες διάγνωσης της ΔΕΠΥ. Επιπλέον, θα ενσωματωθούν υπηρεσίες συνεχόμενης καταγραφής, που όμως θα αφαιρούν την υποκειμενικότητα που εγγενώς υπάρχει στις καταγραφές ειδικών.

Σχετικά με τη μεθοδολογία υλοποίησης του έργου, η πρώτη δραστηριότητα περιλαμβάνει μια έρευνα χαρακτηριστικών για την ΔΕΠΥ. Έπειτα, το παραγόμενο έγγραφο χαρακτηριστικών θα καθοδηγήσει την σχεδίαση και υλοποίηση του παιχνιδιού σοβαρού σκοπού. Κατόπιν, ακολουθούν οι πιλοτικές δοκιμές, όπου θα πραγματοποιηθεί αξιολόγηση της εφαρμογής και παραγωγή δεδομένων. Οι δοκιμές θα γίνουν σε ένα ελεγχόμενο περιβάλλον, που προσομοιάζει πραγματικές συνθήκες (living lab). Με βάση τα δεδομένα και την στατιστική ανάλυση, θα κριθεί εάν τα αποτελέσματα σχετικά με τη διάγνωση είναι επαρκώς καλά, ή αν θα χρειαστεί αναπροσαρμογή του παιχνιδιού.

Παράλληλα, κάποιοι συμμετέχοντες θα βρεθούν σε περισσότερες από μια πιλοτικές δοκιμές, έτσι ώστε να είναι δυνατή η αξιολόγηση της αποτελεσματικότητας της παρέμβασης. Για την αξιολόγηση και την αποτελεσματικότητα των παρεμβάσεων θα χρησιμοποιηθεί η εφαρμογή WHAAM. Τα αποτελέσματα θα γίνονται διαθέσιμα σε ειδικούς, και μόνο αυτοί θα έχουν πλήρη πρόσβαση στα στοιχεία.

Η ADHD360 φιλοδοξεί να αναπτυχθεί σε μία ολοκληρωμένη τεχνολογική πλατφόρμα ΔΕΠΥ και να αποτελέσει το κύριο σημείο αναφοράς για άτομα που θέλουν να πληροφορηθούν ή να χρησιμοποιήσουν τις υπηρεσίες της πλατφόρμας. Το έργο αναμένεται να έχει σημαντική επίδραση στον ευρύτερο πληθυσμό, και συγκεκριμένα σε γονείς και εκπαιδευτικούς, επαγγελματίες υγείας και άτομα με ΔΕΠΥ. Θα υπάρχουν δύο εκδόσεις της εφαρμογής, όπου η πρώτη θα παρέχει διάγνωση ενώ η δεύτερη, διαθέσιμη μέσω συνδρομής, θα περιλαμβάνει περισσότερες δυνατότητες. Το παιχνίδι θα είναι διαθέσιμο σε H/Y μέσω web και στις πλατφόρμες iOS και Android, για χρήση από smartphones και tablets. Σημειώνεται ότι στόχος για την εμπορική αξιοποίηση του ADHD360, πέρα από την Ελληνική αγορά, είναι και οι αγγλόφωνες αγορές . Ακόμα, θα πραγματοποιηθούν δραστηριότητες διάχυσης και δημοσιότητας μέσω online περιεχομένου, διοργάνωση ειδικών εκδηλώσεων πληροφόρησης και συμμετοχή σε συνέδρια και εκδηλώσεις σχετικές με τη ΔΕΠΥ.

Οι φορείς που θα συνεργαστούν για την υλοποίηση του έργου είναι το εργαστήριο SOFTWISE (Πληροφορική ΑΠΘ), το εργαστήριο MEDPHYS (Ιατρική ΑΠΘ) και η εταιρία TSM. Συνδυαστικά, οι φορείς αυτοί καλύπτουν την απαραίτητη τεχνογνωσία σε ζητήματα ανάλυσης δεδομένων, μηχανικής μάθησης, τήρησης ιατρικού πρωτοκόλλου, ανάπτυξης λογισμικού και gamification που απαιτεί το έργο.

# PROPOSAL ABSTRACT

ADΗD is the most commonly diagnosed mental disorder and is mainly characterized by difficulties in paying attention. Up to this day, the diagnosis, the interventions and the evaluation of the treatment effectiveness are based on systematic record of the symptoms done by experts. The systematic reports, even though they add immense value to the diagnosis and treatment of ADHD, are often prone to subjectivity. The source of subjectivity is the fact that these reports are mainly based on qualitative attributes, for instance the answers a child or his parents gives to an expert.

The main objective of this project is the development of a novel, integrated platform, named ADHD360, based on a serious game. The functionality that will be implemented in ADHD360 is a) the diagnosis of the disorder b) the treatment of it’s symptoms through the platform and through interventions and c) the evaluation of their effectiveness.

The interaction of the users with the serious game will produce the necessary data that capture the measurable characteristics of ADHD. The same data will be the input to a series of statistical analyses that will lead to a) the automatic adaptation of the game mechanics for the improvement of diagnostic accuracy and b) the creation of predictive models with Machine Learning methods. These predictive models will be integrated with ADHD360, to provide advanced ADHD diagnosis services. Additionally, ADHD360 will also provide services for continuous record keeping with the least amount of subjectivity.

Regarding project development, the first activity is a study on the features and characteristics of ADHD. The produced document of this activity will guide the design and the implementation of the serious game. A series of test experiments on the game will follow, in order to evaluate the game’s diagnostic effectiveness and to produce validation data. The tests will take place in a controlled environment that simulates realistic conditions (Living Lab).

Any further adjustment and improvement of the game mechanics will be based on an adaptive learning module using reinforcement learning and built right into the ADHD360 platform. Concerning the intervention process, some participants will take part in more than one test experiments (controlled sessions), allowing us to evaluate their effectiveness over time. An appropriately configured version of the WHAMM application will be used and the results will be available exclusively to experts authorized to handle each case.

ADHD360 aspires to develop into an integrated solution that will become the central point for individuals that want to be informed on ADHD or use the provided services. The project is expected to have a significant positive impact on the general population and specifically on of parents, educators, health experts and people with ADHD. The application will come in two versions, the first will provide diagnostic services and the second, available through subscription, will provide more advanced services. The game will be available for PC through the web and for the mobile platforms iOS and Android. It should be noted that ADHD360 focuses on all English and Greek speaking markets. Furthermore, publicity and promotion activities will take place through online content, informational events and the participation in conferences about ADHD.

Τhe SOFTWISE lab (Informatics AUTH), the MEDPHYS lab (Medical School AUTH) and TSM LTD. will cooperate for the development of this project. Together, they have the necessary knowledge and technical expertise regarding data analysis, machine learning, medical protocols, software development and gamification that the project requires.

**ΣΤΟΙΧΕΙΑ ΤΑΥΤΟΤΗΤΑΣ ΔΙΚΑΙΟΥΧΩΝ**

**1.**

**ΓΕΝΙΚΑ ΣΤΟΙΧΕΙΑ ΕΡΓΟΥ ΕΤΑΚ**

**1.1.**

| **ΠΑΡΕΜΒΑΣΗ** | II. Συμπράξεις Επιχειρήσεων με Ερευνητικούς Οργανισμούς |
| --- | --- |
| **ΤΟΜΕΑΣ ΠΡΟΤΕΡΑΙΟΤΗΤΑΣ** | 5-ΥΦΑ: Υγεία και Φάρμακα |
| **ΠΕΡΙΟΧΗ** | 5.5 Ηλεκτρονική Υγεία: Υπηρεσίες και Συστήματα για Ασθενείς/Πολίτες και Επαγγελματίες Υγείας |
| **ΠΡΟΤΕΡΑΙΟΤΗΤΑ** | 5.5.2 Υπηρεσίες και συστήματα για την υποστήριξη εξατομικευμένων προσεγγίσεων αυτοδιαχείρισης χρόνιων ασθενών |
| **ΔΙΑΡΚΕΙΑ (ΜΗΝΕΣ)** | 36 |
| **ΚΑΤΗΓΟΡΙΟΠΟΙΗΣΗ Horizon 2020** | 31053827 |
| **ΛΕΞΕΙΣ ΚΛΕΙΔΙΑ** | ΔΕΠΥ, Σοβαρά Παiχνίδια, Μηχανική Μάθηση, Διαγνωστικά Εργαλεία, Παρέμβαση |
| **KEYWORDS** | ADHD, Serious Games, Machine Learning, Diagnostic Tools, Intervention |

**ΣΤΟΙΧΕΙΑ ΔΙΚΑΙΟΥΧΟΥ ΦΟΡΕΑ (ΦΟΡΕΩΝ ΣΥΜΠΡΑΞΗΣ)**

**1.2.**

**ΣΤΟΙΧΕΙΑ ΤΑΥΤΟΤΗΤΑΣ ΦΟΡΕΑ**

**1.2.1.**

| **1.2.1.1.** | **ΕΙΔΟΣ ΦΟΡΕΑ: ΕΠΙΧΕΙΡΗΣΗ** | |
| --- | --- | --- |
| **ΓΕΝΙΚΑ ΣΤΟΙΧΕΙΑ** | | |
| **Α/Α ΦΟΡΕΑ** | | 3 |
| **ΣΥΝΤΟΝΙΣΤΗΣ** | | Όχι |
| **ΕΠΩΝΥΜΙΑ ΦΟΡΕΑ** | | THE SECOND METHOD ΑΝΩΝΥΜΗ ΕΤΑΙΡΕΙΑ ΚΑΙΝΟΤΟΜΩΝ ΥΠΗΡΕΣΙΩΝ ΕΡΕΥΝΣ ΚΑΙ ΑΝΑΠΤΥΞΗΣ ΕΦΑΡΜΟΓΩΝ ΥΠΟΛΟΓΙΣΤΗ ΚΑΙ ΟΠΤΙΚΟΑΚΟΥΣΤΙΚΗΣ ΠΑΡΑΓΩΓΗΣ |
| **ΣΥΝΤΟΜΟΓΡΑΦΙΑ ΦΟΡΕΑ** | | TSM |
| **ΔΙΑΚΡΙΤΙΚΟΣ ΤΙΤΛΟΣ ΦΟΡΕΑ** | | THE SECOND METHOD ΑΕ |

| **ΣΥΝΟΠΤΙΚΗ ΠΑΡΟΥΣΙΑΣΗ ΔΙΚΑΙΟΥΧΟΥ**  **ΦΟΡΕΑ** | | H The Second Method A.E. ιδρύθηκε τον Οκτώβριο του 2006. Τα γραφεία της είναι στο 9ο χιλ. Θέρμης – Θεσσαλονίκης και συγκεκριμένα στην Θερμοκοιτίδα Επιχειρήσεων THERMI A.E.. Ένας ενεργός R&D τομέας εξειδικεύεται σε εφαρμογές serious games, gamification, Digital media engineering services και advanced internet applications Στα Serious Games η εταιρία επικεντρώνεται στην ανάπτυξη παιχνιδιών για την υγεία. Κυκλοφορεί στην Ελληνική και Κυπριακή αγορά τη σειρά PLAY “Λογισμικό Λογοθερπείας» . Το λογισμικό βοηθάει τον θεραπευτή να καθοδηγήσει το παιδί στην κατάκτηση του φωνήματος μέσα από ευχάριστες διαδραστικές διαδικασίες. Η εταιρεία έχει αναπτύξει σειρά εκπαιδευτικών εφαρμογών για smart phones. Προσφέρει εξιδικευμένες υπηρεσίες εφαρμόζοντας νέες προσεγγίσεις στην διάδραση ανθρώπου – υπολογιστών με την χρήση αισθητήρων (ambient intelligence) και video mapping.  Στο διάστημα της λειτουργίας της η εταιρία έχει συμμετοχή σε ερευνητικά προγράμματα και έχει αναπτύξει συνεργασίες με ερευνητικά ινστιτούτα, Πανεπιστημιακά ιδρύματα, και μουσεία επιστήμης και τεχνολογίας. |
| --- | --- | --- |
| **Α.Φ.Μ.** | | 998755755 |
| **Δ.Ο.Υ.** | | ΘΕΣΣΑΛΟΝΙΚΗΣ ΦΑΕ |
| **ΧΩΡΑ** | | ΕΛΛΑΔΑ |
| **ΗΜΕΡΟΜΗΝΙΑ ΙΔΡΥΣΗΣ** | | 08/05/2006 |
| **ΝΟΜΙΚΗ ΜΟΡΦΗ** | | Ανώνυμη Εταιρεία (Α.Ε) |
| **ΜΕΓΕΘΟΣ ΕΠΙΧΕΙΡΗΣΗΣ** | | Πολύ Μικρή |
| **ΔΙΕΥΘΥΝΣΗ ΕΔΡΑΣ** | | |
| **ΠΕΡΙΦΕΡΕΙΑ** | | Κεντρικής Μακεδονίας |
| **ΠΕΡΙΦΕΡΕΙΑΚΗ ΕΝΟΤΗΤΑ** | | ΘΕΣΣΑΛΟΝΙΚΗΣ |
| **ΔΗΜΟΣ** | | Πυλαίας - Χορτιάτη |
| **ΔΗΜΟΤΙΚΗ-ΤΟΠΙΚΗ ΚΟΙΝΟΤΗΤΑ** | | ΔΗΜΟΤΙΚΗ ΕΝΟΤΗΤΑ ΠΥΛΑΙΑΣ ΔΗΜΟΤΙΚΗ ΚΟΙΝΟΤΗΤΑ ΠΥΛΑΙΑΣ |
| **ΔΙΕΥΘΥΝΣΗ** | **ΟΔΟΣ – ΑΡΙΘΜΟΣ** | ΣΤ. ΚΑΖΑΝΤΖΙΔΗ 47 |
|  | **ΤΑΧ. ΚΩΔΙΚΟΣ** | 57001 |
|  | **ΠΟΛΗ** | ΘΕΣΣΑΛΟΝΙΚΗ |
| **ΤΗΛΕΦΩΝΟ ΕΠΙΚΟΙΝΩΝΙΑΣ** | | 2311999999 |
| **FAX** | | 2311999997 |
| **Ιστοχώρος (Website)** | | <http://www.thesecondmethod.com/> |
| **Ηλεκτρονική Διεύθυνση (e-mail)** | | [c.karapiperis@thesecondmethod.com](mailto:c.karapiperis@thesecondmethod.com) |
| **Άλλη Ηλεκτρονική Διεύθυνση**  **(e-mail)** | | [eirini@thermi-group.com](mailto:eirini@thermi-group.com) |

**ΕΙΔΟΣ ΦΟΡΕΑ: Λοιποί Φορείς που αντιμετωπίζονται ως Επιχειρήσεις εάν ασκούν οικονομική δραστηριότητα που συνίσταται στην προσφορά προϊόντων ή υπηρεσιών σε δεδομένη αγορά**

**1.2.1.2.**

| **1.2.1.3.** | **ΕΙΔΟΣ ΦΟΡΕΑ: ΕΡΕΥΝΗΤΙΚΟΣ ΟΡΓΑΝΙΣΜΟΣ** | |
| --- | --- | --- |
| **ΓΕΝΙΚΑ ΣΤΟΙΧΕΙΑ** | | |
| **Α/Α ΦΟΡΕΑ** | | 1 |
| **ΣΥΝΤΟΝΙΣΤΗΣ** | | Ναι |

| **ΕΠΩΝΥΜΙΑ ΦΟΡΕΑ** | | Εργαστήριο Τεχνολογίας Λογισμικού, Ιστού και Ευφυών Συστημάτων – ΤΜΗΜΑ Πληροφορικής-ΑΠΘ |
| --- | --- | --- |
| **ΣΥΝΤΟΜΟΓΡΑΦΙΑ ΦΟΡΕΑ** | | SOFTWISE |
| **ΕΠΩΝΥΜΙΑ ΚΥΡΙΟΥ ΦΟΡΕΑ ΣΤΟΝ**  **ΟΠΟΙΟ ΑΝΗΚΕΙ** | | Αριστοτέλειο Πανεπιστήμιο Θεσσαλονίκης - Ειδικός Λογαριασμός Κονδυλίων Έρευνας |
| **ΣΥΝΟΠΤΙΚΗ ΠΑΡΟΥΣΙΑΣΗ ΔΙΚΑΙΟΥΧΟΥ ΦΟΡΕΑ** | | Το Εργαστήριο Τεχνολογίας Λογισμικού, Ιστού και Ευφυών Συστημάτων (SOFTWISE) του Τμήματος Πληροφορικής του Αριστοτέλειου Πανεπιστημίου Θεσσαλονίκης ιδρύθηκε το Φεβρουάριο του 2017 ως συνέχεια του εργαστηρίου Γλωσσών Προγραμματισμού και Τεχνολογίας Λογισμικού που υπήρχε στο Τμήμα από τον Ιανουάριο του 1998. Το εργαστήριο διευθύνεται από τον Καθηγητή κ. Ιωάννη Βλαχάβα, ο οποίος διατελεί και πρόεδρος του Τμήματος Πληφορορικής του ΑΠΘ, αποτελείται από επτά μέλη ΔΕΠ, πάνω από είκοσι υποψήφιους διδάκτορες και δέκα μεταδιδακτορικούς ερευνητές – εξωτερικούς συνεργάτες. Το εργαστήριο SOFTWISE καλύπτει εκπαιδευτικές και ερευνητικές ανάγκες στα ακόλουθα πεδία:   - Ευφυή Συστήματα και Επεξεργασία Γνώσης - Μηχανική Μάθηση και Ανακάλυψη Γνώσης - Τεχνολογία Λογισμικού - Λειτουργικά συστήματα - Στατιστική και Επιχειρησιακή Έρευνα   Το εργαστήριο έχει συμμετάσχει σε περισσότερα από 40 ερευνητικά και αναπτυξιακά προγράμματα στα προαναφερθέντα ερευνητικά αντικείμενα και τα μέλη του έχουν δημοσιεύσει περισσότερες από 500 ερευνητικές εργασίες σε συνέδρια και περιοδικά του χώρου.  Επιπλέον του ερευνητικού και διδακτικού του ρόλου, το εργαστήριο SOFTWISE δραστηριοποιείται με επιτυχία στην Παραγωγή Συστημάτων διαθέτοντας πιστοποιημένο Σύστημα Διαχείρισης Ποιότητας σύμφωνα με τις απαιτήσεις του Διεθνούς Προτύπου ISO 9001:2008. |
| **Α.Φ.Μ.** | | 090049627 |
| **Δ.Ο.Υ.** | | ΘΕΣΣΑΛΟΝΙΚΗΣ Δ' |
| **ΧΩΡΑ** | | ΕΛΛΑΔΑ |
| **ΚΥΡΙΑ ΔΡΑΣΤΗΡΙΟΤΗΤΑ ΕΡΕΥΝΗΤΙΚΟΥ ΟΡΓΑΝΙΣΜΟΥ** | | Δραστηριότητες εκπαίδευσης για την εξασφάλιση περισσότερων και πιο ειδικευμένων ανθρώπινων πόρων |
| **ΕΑΝ Η ΑΠΑΝΤΗΣΗ ΣΤΗΝ ΠΡΟΗΓΟΥΜΕΝΗ ΕΡΩΤΗΣΗ ΕΙΝΑΙ**  **«ΑΛΛΗ» ΠΡΟΣΔΙΟΡΙΣΤΕ:** | |  |
| **ΔΙΕΥΘΥΝΣΗ ΕΔΡΑΣ** | | |
| **ΠΕΡΙΦΕΡΕΙΑ** | | Κεντρικής Μακεδονίας |
| **ΠΕΡΙΦΕΡΕΙΑΚΗ ΕΝΟΤΗΤΑ** | | ΘΕΣΣΑΛΟΝΙΚΗΣ |
| **ΔΗΜΟΣ** | | Θεσσαλονίκης |
| **ΔΗΜΟΤΙΚΗ-ΤΟΠΙΚΗ ΚΟΙΝΟΤΗΤΑ** | | ΔΗΜΟΤΙΚΗ ΕΝΟΤΗΤΑ ΘΕΣΣΑΛΟΝΙΚΗΣ ΔΗΜΟΤΙΚΗ ΚΟΙΝΟΤΗΤΑ 1ου ΔΗΜ.ΔΙΑΜΕΡ.ΘΕΣΣΑΛΟΝΙΚΗΣ |
| **ΔΙΕΥΘΥΝΣΗ** | **ΟΔΟΣ – ΑΡΙΘΜΟΣ** | Κτίριο ΚΕ.Δ.Ε.Α. 3ης Σεπτεμβρίου – Πανεπιστημιούπολη |
|  | **ΤΑΧ. ΚΩΔΙΚΟΣ** | ΘΕΣΣΑΛΟΝΙΚΗ |
|  | **ΠΟΛΗ** | 54636 |
| **Ιστοχώρος (Website)** | | [http://softwise.csd.auth.gr](http://softwise.csd.auth.gr/) |

| **ΓΕΝΙΚΑ ΣΤΟΙΧΕΙΑ** | |
| --- | --- |
| **Α/Α ΦΟΡΕΑ** | 2 |
| **ΣΥΝΤΟΝΙΣΤΗΣ** | Όχι |

| **ΕΠΩΝΥΜΙΑ ΦΟΡΕΑ** | | ΕΡΓΑΣΤΗΡΙΟ ΙΑΤΡΙΚΗΣ ΦΥΣΙΚΗΣ – ΤΜΗΜΑ ΙΑΤΡΙΚΗΣ ΑΠΘ |
| --- | --- | --- |
| **ΣΥΝΤΟΜΟΓΡΑΦΙΑ ΦΟΡΕΑ** | | MEDPHYS |
| **ΕΠΩΝΥΜΙΑ ΚΥΡΙΟΥ ΦΟΡΕΑ ΣΤΟΝ**  **ΟΠΟΙΟ ΑΝΗΚΕΙ** | | ΕΙΔΙΚΟΣ ΛΟΓΑΡΙΑΣΜΟΣ ΚΟΝΔΥΛΙΩΝ ΕΡΕΥΝΑΣ ΑΠΘ |
| **ΣΥΝΟΠΤΙΚΗ ΠΑΡΟΥΣΙΑΣΗ ΔΙΚΑΙΟΥΧΟΥ ΦΟΡΕΑ** | | Το Εργαστήριο Ιατρικής Φυσικής της Ιατρικής Σχολής του Αριστοτελείου Πανεπιστημίου Θεσσαλονίκης θεμελιώθηκε το 1964, το πρώτο εργαστήριο Ιατρικής Φυσικής στην ελληνική Τριτοβάθμια Εκπαίδευση. Η αποστολή του Εργαστηρίου είναι να συμβάλλει στην κατάρτιση και τη διεπιστημονική έρευνα σε ένα περιβάλλον που ευνοεί τη δημιουργικότητα και συνέργεια. Το Εργαστήριο εξειδικεύεται στην εκπαίδευση νέων ιατρών στις αρχές της φυσικής, της βιοϊατρικής τεχνολογίας και των εφαρμογών τους στις Επιστήμες Υγείας μέσω των μαθημάτων της Ιατρικής Φυσικής και Βιοϊατρικής Τεχνολογίας, καθώς και στην Ιατρική Εκπαίδευση στα μεταπτυχιακά προγράμματα της Σχολής Επιστημών Υγείας. Το Εργαστήριο συμμετέχει επίσης στο Διαπανεπιστημιακό μεταπτυχιακό πρόγραμμα της Ιατρικής Φυσικής (Radiophysics), καθώς και, στην Συνεχιζόμενη Ιατρική Εκπαίδευση (ΣΙΕ) στην ακτινολογία, την ακτινοθεραπεία και την πυρηνική ιατρική.  Το εργαστήριο είναι μια δυναμική, διαδραστική κοινότητα όπου συμμετέχουν 14 διδακτορικοί φοιτητές, 25 επιστημονικοί συνεργάτες και 2 μέλη ΔΕΠ, οι οποίοι εργάζονται ως πρωτοπόροι σε ερευνητικά πεδία όπως Εφαρμοσμένες Νευροεπιστήμες, Ακτινοδιαγνωστική  και μη ιοντίζουσες ακτινοβολίες, Ιατρική εκπαίδευση, Υποστηρικτικές τεχνολογίες, Ιατρική απεικόνισης, Σημασιολογικός Ιστός, Συναισθηματική υπολογιστική και άλλους σύγχρονους θεματικούς τομείς. Αποτελείται από 10 ερευνητικές ομάδες οι οποίες επιδιώκουν  καινοτόμα ερευνητικά προγράμματα. Έχουν αναγνωριστεί διεθνώς για την αριστεία τους στην έρευνα και έχουν χρηματοδοτηθεί από ένα ευρύ φάσμα πηγών όπως το 6ο ΠΠ, το 7ο ΠΠ, INTERREG, ΠΡΟΓΡΑΜΜΑ ΔΙΑ ΒΙΟΥ ΜΑΘΗΣΗΣ, εθνικοί πόροι από το Υπουργείο  Παιδείας, Υπουργείο Υγείας, Γενική Γραμματεία Έρευνας και Τεχνολογίας και άλλους εθνικούς και διεθνείς φορείς.  Επίσης, εξειδικεύεται στην παραγωγή λογισμικού και διαθέτει ISO 9001. |
| **Α.Φ.Μ.** | | 090049627 |
| **Δ.Ο.Υ.** | | ΘΕΣΣΑΛΟΝΙΚΗΣ Δ' |
| **ΧΩΡΑ** | | ΕΛΛΑΔΑ |
| **ΚΥΡΙΑ ΔΡΑΣΤΗΡΙΟΤΗΤΑ ΕΡΕΥΝΗΤΙΚΟΥ ΟΡΓΑΝΙΣΜΟΥ** | | Δραστηριότητες εκπαίδευσης για την εξασφάλιση περισσότερων και πιο ειδικευμένων ανθρώπινων πόρων |
| **ΕΑΝ Η ΑΠΑΝΤΗΣΗ ΣΤΗΝ ΠΡΟΗΓΟΥΜΕΝΗ ΕΡΩΤΗΣΗ ΕΙΝΑΙ**  **«ΑΛΛΗ» ΠΡΟΣΔΙΟΡΙΣΤΕ:** | |  |
| **ΔΙΕΥΘΥΝΣΗ ΕΔΡΑΣ** | | |
| **ΠΕΡΙΦΕΡΕΙΑ** | | Κεντρικής Μακεδονίας |
| **ΠΕΡΙΦΕΡΕΙΑΚΗ ΕΝΟΤΗΤΑ** | | ΘΕΣΣΑΛΟΝΙΚΗΣ |
| **ΔΗΜΟΣ** | | Θεσσαλονίκης |
| **ΔΗΜΟΤΙΚΗ-ΤΟΠΙΚΗ ΚΟΙΝΟΤΗΤΑ** | | ΔΗΜΟΤΙΚΗ ΕΝΟΤΗΤΑ ΘΕΣΣΑΛΟΝΙΚΗΣ ΔΗΜΟΤΙΚΗ ΚΟΙΝΟΤΗΤΑ 1ου ΔΗΜ.ΔΙΑΜΕΡ.ΘΕΣΣΑΛΟΝΙΚΗΣ |
| **ΔΙΕΥΘΥΝΣΗ** | **ΟΔΟΣ – ΑΡΙΘΜΟΣ** | Κτίριο ΚΕ.Δ.Ε.Α. 3ης Σεπτεμβρίου – Πανεπιστημιούπολη |
|  | **ΤΑΧ. ΚΩΔΙΚΟΣ** | ΘΕΣΣΑΛΟΝΙΚΗ |
|  | **ΠΟΛΗ** | 54636 |
| **Ιστοχώρος (Website)** | | [http://medpys.med.auth.gr](http://medpys.med.auth.gr/) |

**ΕΙΔΟΣ ΦΟΡΕΑ: Λοιποί Φορείς που αντιμετωπίζονται ως Ερευνητικοί Οργανισμοί**

**1.2.1.4.**

**ΒΑΣΙΚΑ ΣΤΟΙΧΕΙΑ ΤΟΠΟΥ ΥΛΟΠΟΙΗΣΗΣ ΕΡΓΟΥ**

**/ ΣΤΟΙΧΕΙΑ ΠΑΡΑΡΤΗΜΑΤΟΣ ΤΟ ΟΠΟΙΟ ΥΛΟΠΟΙΕΙ ΤΟ ΕΡΓΟ**

**1.2.2.**

| **ΦΟΡΕΑΣ** | | SOFTWISE |
| --- | --- | --- |
| **ΠΕΡΙΦΕΡΕΙΑ** | | Κεντρικής Μακεδονίας |
| **ΠΕΡΙΦΕΡΕΙΑΚΗ ΕΝΟΤΗΤΑ** | | ΘΕΣΣΑΛΟΝΙΚΗΣ |
| **ΔΗΜΟΣ** | | Θεσσαλονίκης |
| **ΔΗΜΟΤΙΚΗ-ΤΟΠΙΚΗ ΚΟΙΝΟΤΗΤΑ** | | ΔΗΜΟΤΙΚΗ ΕΝΟΤΗΤΑ ΘΕΣΣΑΛΟΝΙΚΗΣ ΔΗΜΟΤΙΚΗ ΚΟΙΝΟΤΗΤΑ 1ου ΔΗΜ.ΔΙΑΜΕΡ.ΘΕΣΣΑΛΟΝΙΚΗΣ |
| **ΔΙΕΥΘΥΝΣΗ** | **ΟΔΟΣ – ΑΡΙΘΜΟΣ** | Κτίριο ΚΕ.Δ.Ε.Α. 3ης Σεπτεμβρίου – Πανεπιστημιούπολη |
|  | **ΤΑΧ. ΚΩΔΙΚΟΣ** | 54636 |
|  | **ΠΟΛΗ** | Θεσσαλονίκη |
| **ΤΗΛΕΦΩΝΟ ΕΠΙΚΟΙΝΩΝΙΑΣ** | | 2310998145 |
| **FAX** | | 2310998362 |
| **e-mail** | | [vlahavas@csd.auth.gr](mailto:vlahavas@csd.auth.gr) |

| **ΦΟΡΕΑΣ** | | MEDPHYS |
| --- | --- | --- |
| **ΠΕΡΙΦΕΡΕΙΑ** | | Κεντρικής Μακεδονίας |
| **ΠΕΡΙΦΕΡΕΙΑΚΗ ΕΝΟΤΗΤΑ** | | ΘΕΣΣΑΛΟΝΙΚΗΣ |
| **ΔΗΜΟΣ** | | Θεσσαλονίκης |
| **ΔΗΜΟΤΙΚΗ-ΤΟΠΙΚΗ ΚΟΙΝΟΤΗΤΑ** | | ΔΗΜΟΤΙΚΗ ΕΝΟΤΗΤΑ ΘΕΣΣΑΛΟΝΙΚΗΣ ΔΗΜΟΤΙΚΗ ΚΟΙΝΟΤΗΤΑ 1ου ΔΗΜ.ΔΙΑΜΕΡ.ΘΕΣΣΑΛΟΝΙΚΗΣ |
| **ΔΙΕΥΘΥΝΣΗ** | **ΟΔΟΣ – ΑΡΙΘΜΟΣ** | Κτίριο ΚΕ.Δ.Ε.Α. 3ης Σεπτεμβρίου – Πανεπιστημιούπολη |
|  | **ΤΑΧ. ΚΩΔΙΚΟΣ** | 54636 |
|  | **ΠΟΛΗ** | Θεσσαλονίκη |
| **ΤΗΛΕΦΩΝΟ ΕΠΙΚΟΙΝΩΝΙΑΣ** | | 2310999237 |
| **FAX** | | 2310999702 |
| **e-mail** | | [bamidis@med.auth.gr](mailto:bamidis@med.auth.gr) |

| **ΦΟΡΕΑΣ** | | TSM |
| --- | --- | --- |
| **ΠΕΡΙΦΕΡΕΙΑ** | | Κεντρικής Μακεδονίας |
| **ΠΕΡΙΦΕΡΕΙΑΚΗ ΕΝΟΤΗΤΑ** | | ΘΕΣΣΑΛΟΝΙΚΗΣ |
| **ΔΗΜΟΣ** | | Πυλαίας - Χορτιάτη |
| **ΔΗΜΟΤΙΚΗ-ΤΟΠΙΚΗ ΚΟΙΝΟΤΗΤΑ** | | ΔΗΜΟΤΙΚΗ ΕΝΟΤΗΤΑ ΠΥΛΑΙΑΣ ΔΗΜΟΤΙΚΗ ΚΟΙΝΟΤΗΤΑ ΠΥΛΑΙΑΣ |
| **ΔΙΕΥΘΥΝΣΗ** | **ΟΔΟΣ – ΑΡΙΘΜΟΣ** | ΣΤ. ΚΑΖΑΝΤΖΙΔΗ 47 |
|  | **ΤΑΧ. ΚΩΔΙΚΟΣ** | 57001 |
|  | **ΠΟΛΗ** | ΘΕΣΣΑΛΟΝΙΚΗ |
| **ΤΗΛΕΦΩΝΟ ΕΠΙΚΟΙΝΩΝΙΑΣ** | | 2311999999 |

| **FAX** | 2311999997 |
| --- | --- |
| **e-mail** | [c.karapiperis@thesecondmethod.com](mailto:c.karapiperis@thesecondmethod.com) |

**ΑΞΙΟΛΟΓΗΣΗ ΘΕΜΑΤΙΚΟΥ ΤΟΜΕΑ**

**2.**

| **ΑΞΙΟΛΟΓΗΣΗ ΤΟΜΕΑ ΠΡΟΤΕΡΑΙΟΤΗΤΑΣ** | |
| --- | --- |
| **Αριθμός Μητρώου Χρήστη** | 12648 |
| **Η πρόταση εμπίπτει στον Τομέα προτεραιότητας που έχει δηλωθεί;** | Όχι |
| **Αν προτείνετε αλλαγή Τομέα προτεραιότητας, παρακαλούμε επιλέξτε τον Τομέα προτεραιότητας που ταιριάζει με**  **το προτεινόμενο έργο** | 5-ΥΦΑ: Υγεία και Φάρμακα |

| **2Η ΑΞΙΟΛΟΓΗΣΗ ΤΟΜΕΑ ΠΡΟΤΕΡΑΙΟΤΗΤΑΣ** | |
| --- | --- |
| **Η πρόταση εμπίπτει στον Τομέα προτεραιότητας που έχει δηλωθεί;** | Ναι |
| **Τεκμηρίωση** | . |

**ΚΡΙΤΗΡΙΑ ΑΞΙΟΛΟΓΗΣΗΣ**

**3.**

| **Κριτήριο** | **Τεκμηρίωση** | **Βαθμολογία** |
| --- | --- | --- |
| **Κριτήριο Α.**  **Επιστημονική και τεχνική αρτιότητα του προτεινόμενου έργου (Excellence)** | Η πρόταση ADHD360 αφορά στη ανάπτυξη μίας ολοκληρωμένης τεχνολογικής πλατφόρμας, με στόχο τη διάγνωση της Διαταραχής Ελλειμματικής Προσοχής και Υπερκινητικότητας (ΔΕΠΥ), την αντιμετώπιση των συμπτωμάτων μέσω της ίδιας της εφαρμογής αλλά και οργανωμένων παρεμβάσεων της και την αξιολόγηση της αποτελεσματικότητας αυτών. Κεντρικό στοιχείο της πλατφόρμας θα είναι ένα παιχνίδι σοβαρού σκοπού (serious game) Προκύπτει συνάφεια με τους στόχους της προκήρυξης στον τομέα της ηλεκτρονικής υγείας αν και δεν τεκμηριώνεται σαφώς η συσχέτιση με κάποια από τις δραστηριότητές του τομέα - με την προτεινόμενη ένταξη στην θεματική ενότητα για «ανάπτυξη ψηφιακών παιγνιδιών»  Δεν αναλύεται επαρκώς η τρέχουσα τεχνολογική στάθμη και κυρίως δεν τεκμηριώνεται εάν και με ποιο τρόπο το | 3 |

|  | προτεινόμενο έργο εκτείνεται πέρα της τρέχουσας τεχνολογικής στάθμης. |  |
| --- | --- | --- |
| **Κριτήριο Β.**  **Εμπειρία και αξιοπιστία του (ων) δικαιούχου (ων) και ποιότητα και ικανότητα του τρόπου υλοποίησης του έργου (Implementation)** | Το σχέδιο εργασίας είναι συνεκτικό και αποτελεσματικό στην υλοποίηση του έργου και η ικανότητα των δικαιούχων κρίνεται επαρκής.  Δεν περιγράφεται αναλυτικά ο τρόπος εκτέλεσης πιλοτικών δοκιμών οι οποίες σημειωτέον θα γίνουν μόνο σε περιβάλλον προσομοίωσης (living lab) Επιπλέον, η απουσία κλινικών εταίρων που θα υποστήριζαν τις πιλοτικές δοκιμές αξιολογείται αρνητικά | 3 |
| **Κριτήριο Γ. Αποτελέσματα και επιπτώσεις του προτεινόμενου σχεδίου (Impact)** | Δεν τεκμηριώνεται επαρκώς η συνεισφορά στην αριστεία και της ανταγωνιστικότητας της οικονομίας σε εθνικό επίπεδο.  Δεν τεκμηριώνεται η αποτελεσματικότητα των προτεινόμενων σχεδίων εκμετάλλευσης και διάχυσης των αποτελεσμάτων του έργου (π.χ. δεδομένου ότι δεν εμφανίζονται δαπάνες συμμετοχής σε εκθέσεις). | 3 |

**ΟΙΚΟΝΟΜΙΚΑ ΣΤΟΙΧΕΙΑ ΤΟΥ ΕΡΓΟΥ**

**4.**

**ΑΝΑΛΥΣΗ ΤΟΥ ΠΡΟΫΠΟΛΟΓΙΣΜΟΥ ΑΝΑ ΦΟΡΕΑ ΚΑΙ ΚΑΤΗΓΟΡΙΑ ΔΑΠΑΝΗΣ**

**4.1.**

**4.1.1. ΚΑΤΑ ΤΗΝ ΥΠΟΒΟΛΗ**

| **ΕΡ1 - Δαπάνες προσωπικού** | | | | | | |
| --- | --- | --- | --- | --- | --- | --- |
| Α/Α | Φορέας | Περιγραφή | Ειδικότητα | Τιμή Α/μήνα | Α/μήνες | Συνολική Δαπάνη |
|  | TSM |  | 1 R&D Director TSM, 1 Product Manager, 1 Lead Programmer TSM, 1 2D /3D Graphics Designer TSM, 1 Sound Designer | 2.500,00 € | 88 | 220.000,00 € |
|  | SOFTWISE |  | 3 μέλη ΔΕΠ Πληροφορικής, 1  Μεταδιδάκτωρ ερυνητής, 2 Υποψήφιοι Διδάκτορες στην Πληροφορική, 3 Μεταπτυχιακοί φοιτητές | 2.700,00 € | 68,9 | 186.030,00 € |
|  | MEDPHYS |  | 1 Μέλος ΔΕΠ Ιατρικής, 1  Μεταδιδάκτωρ ερευνητής, 3  Υποψήφιοι Διδάκτορες, 3 Μεταπτυχιακοί φοιτητές | 2.500,00 € | 68,4 | 171.000,00 € |

| **ΕΡ2 - Δαπάνες οργάνων και εξοπλισμού, κτιρίων και γηπέδων** | | | | | | | | |
| --- | --- | --- | --- | --- | --- | --- | --- | --- |
| Α/Α | Φορέας | Περιγραφή | Τεκμηρίωση Αναγκαιότητας | Τύπος/Μοντέλο | Έτος Κτήσης | Κόστος Αγοράς | Διάρκεια Απόσβεσης (Μήνες) | Αξία Απόσβεσης |

| **ΕΡ3 - Δαπάνες για έρευνα επί συμβάσει, γνώσεις και διπλώματα ευρεσιτεχνίας, συμβουλευτικές υπηρεσίες** | | | | | |
| --- | --- | --- | --- | --- | --- |
| Α/Α | Φορέας | Περιγραφή | Τεκμηρίωση Αναγκαιότητας | Επωνυμία Προμηθευτή / Μελετητή | Δαπάνη |
|  | TSM | TBC | TBC | TBC | 4.000,00 € |

| **ΕΡ4 - Πρόσθετα γενικά έξοδα και λοιπές λειτουργικές δαπάνες** | | | |
| --- | --- | --- | --- |
| Α/Α | Φορέας | Περιγραφή | Δαπάνη |

|  | TSM | Λειτουργικές δαπάνες, μετακινήσεις, συνέδρια, "Cloud" υπηρεσίες | 28.000,00 € |
| --- | --- | --- | --- |
|  | SOFTWISE | Λειτουργικές δαπάνες, μετακινήσεις, συνέδρια | 5.000,00 € |
|  | MEDPHYS | Λειτουργικές δαπάνες, μετακινήσεις, συνέδρια | 5.000,00 € |

| **ΕΜΕΟ - Έμμεσες λειτουργικές δαπάνες** | | | |
| --- | --- | --- | --- |
| Α/Α | Φορέας | Περιγραφή | Δαπάνη |
|  | SOFTWISE | Έμμεσες Λειτουργικές δαπάνες | 23.951,00 € |
|  | MEDPHYS | Έμμεσες Λειτουργικές δαπάνες | 21.730,00 € |

| **ΜΕ1 - Δαπάνες για μελέτες τεχνικής σκοπιμότητας** | | | |
| --- | --- | --- | --- |
| Α/Α | Φορέας | Περιγραφή | Δαπάνη |

| **ΚΑ1 - Δαπάνες για ενισχύσεις καινοτομίας για ΜΜΕ** | | | |
| --- | --- | --- | --- |
| Α/Α | Φορέας | Περιγραφή | Δαπάνη |

| **ΕΚ1 - Δαπάνες συμμετοχής ΜΜΕ σε εμπορικές εκθέσεις** | | | |
| --- | --- | --- | --- |
| Α/Α | Φορέας | Περιγραφή | Δαπάνη |

**4.1.2. ΚΑΤΑ ΤΗΝ ΑΞΙΟΛΟΓΗΣΗ**

| **ΕΡ1 - Δαπάνες προσωπικού** | | | | | | |
| --- | --- | --- | --- | --- | --- | --- |
| Α/Α | Φορέας | Περιγραφή | Ειδικότητα | Τιμή Α/μήνα | Α/μήνες | Συνολική Δαπάνη |
|  | TSM |  | 1 R&D Director TSM, 1 Product Manager, 1 Lead Programmer TSM, 1 2D /3D Graphics Designer TSM, 1 Sound Designer | 2.500,00 € | 88 | 220.000,00 € |
|  | SOFTWISE |  | 3 μέλη ΔΕΠ Πληροφορικής, 1  Μεταδιδάκτωρ ερυνητής, 2 Υποψήφιοι Διδάκτορες στην Πληροφορική, 3 Μεταπτυχιακοί | 2.700,00 € | 68,9 | 186.030,00 € |

|  |  |  | φοιτητές |  |  |  |
| --- | --- | --- | --- | --- | --- | --- |
|  | MEDPHYS |  | 1 Μέλος ΔΕΠ Ιατρικής, 1  Μεταδιδάκτωρ ερευνητής, 3  Υποψήφιοι Διδάκτορες, 3 Μεταπτυχιακοί φοιτητές | 2.500,00 € | 68,4 | 171.000,00 € |

| **ΕΡ2 - Δαπάνες οργάνων και εξοπλισμού, κτιρίων και γηπέδων** | | | | | | | | |
| --- | --- | --- | --- | --- | --- | --- | --- | --- |
| Α/Α | Φορέας | Περιγραφή | Τεκμηρίωση Αναγκαιότητας | Τύπος/Μοντέλο | Έτος Κτήσης | Κόστος Αγοράς | Διάρκεια Απόσβεσης (Μήνες) | Αξία Απόσβεσης |

| **ΕΡ3 - Δαπάνες για έρευνα επί συμβάσει, γνώσεις και διπλώματα ευρεσιτεχνίας, συμβουλευτικές υπηρεσίες** | | | | | |
| --- | --- | --- | --- | --- | --- |
| Α/Α | Φορέας | Περιγραφή | Τεκμηρίωση Αναγκαιότητας | Επωνυμία Προμηθευτή / Μελετητή | Δαπάνη |
|  | TSM | TBC | TBC | TBC | 4.000,00 € |

| **ΕΡ4 - Πρόσθετα γενικά έξοδα και λοιπές λειτουργικές δαπάνες** | | | |
| --- | --- | --- | --- |
| Α/Α | Φορέας | Περιγραφή | Δαπάνη |
|  | TSM | Λειτουργικές δαπάνες, μετακινήσεις, συνέδρια, "Cloud" υπηρεσίες | 28.000,00 € |
|  | SOFTWISE | Λειτουργικές δαπάνες, μετακινήσεις, συνέδρια | 5.000,00 € |
|  | MEDPHYS | Λειτουργικές δαπάνες, μετακινήσεις, συνέδρια | 5.000,00 € |

| **ΕΜΕΟ - Έμμεσες λειτουργικές δαπάνες** | | | |
| --- | --- | --- | --- |
| Α/Α | Φορέας | Περιγραφή | Δαπάνη |
|  | SOFTWISE | Έμμεσες Λειτουργικές δαπάνες | 23.951,00 € |
|  | MEDPHYS | Έμμεσες Λειτουργικές δαπάνες | 21.730,00 € |

| **ΜΕ1 - Δαπάνες για μελέτες τεχνικής σκοπιμότητας** | | | |
| --- | --- | --- | --- |
| Α/Α | Φορέας | Περιγραφή | Δαπάνη |

| **ΚΑ1 - Δαπάνες για ενισχύσεις καινοτομίας για ΜΜΕ** | | | |
| --- | --- | --- | --- |
| Α/Α | Φορέας | Περιγραφή | Δαπάνη |

| **ΕΚ1 - Δαπάνες συμμετοχής ΜΜΕ σε εμπορικές εκθέσεις** | | | |
| --- | --- | --- | --- |
| Α/Α | Φορέας | Περιγραφή | Δαπάνη |

**ΠΡΟΫΠΟΛΟΓΙΣΜΟΣ ΑΝΑ ΦΟΡΕΑ, ΕΝΟΤΗΤΑ ΕΡΓΑΣΙΑΣ ΚΑΙ ΚΑΤΗΓΟΡΙΑ ΔΡΑΣΤΗΡΙΟΤΗΤΑΣ**

**4.2.**

**4.2.1. ΚΑΤΑ ΤΗΝ ΥΠΟΒΟΛΗ**

| **ΦΟΡΕΑΣ** | **ΕΝΟΤΗΤΑ ΕΡΓΑΣΙΑΣ** | **ΚΑΤΗΓΟΡΙΑ ΔΡΑΣΤΗΡΙΟΤΗΤΑΣ** | **ΠΡΟΥΠΟΛΟΓΙΣΜΟΣ** | **ΔΗΜΟΣΙΑ ΔΑΠΑΝΗ** | **ΕΝΤΑΣΗ ΕΝΙΣΧΥΣΗΣ** |
| --- | --- | --- | --- | --- | --- |
| TSM | Απαιτήσεις χρηστών, αρχιτεκτονική συστήματος και προδιαγραφές | Βιομηχανική Έρευνα (Άρθρο 25) | 62.500,00 € | 50.000,00 € | 80,00 |
| TSM | Ενσωμάτωση απαιτήσεων, υλοποίηση και έλεγχος συστήματος | Βιομηχανική Έρευνα (Άρθρο 25) | 97.500,00 € | 78.000,00 € | 80,00 |
| TSM | Πιλοτικές δοκιμές, αξιολόγηση και προσαρμογή εφαρμογής | Πειραματική Ανάπτυξη (Άρθρο 25) | 45.000,00 € | 27.000,00 € | 60,00 |
| TSM | Αξιοποίηση αποτελεσμάτων | Βιομηχανική Έρευνα (Άρθρο 25) | 47.000,00 € | 37.600,00 € | 80,00 |
| SOFTWISE | Απαιτήσεις χρηστών, αρχιτεκτονική συστήματος και προδιαγραφές | Βιομηχανική Έρευνα (Άρθρο 25) | 49.187,75 € | 49.187,75 € | 100,00 |
| SOFTWISE | Ενσωμάτωση απαιτήσεων, υλοποίηση και έλεγχος συστήματος | Βιομηχανική Έρευνα (Άρθρο 25) | 51.887,75 € | 51.887,75 € | 100,00 |
|  | Πιλοτικές δοκιμές, αξιολόγηση και | Πειραματική Ανάπτυξη (Άρθρο 25) | 68.087,75 € | 68.087,75 € |  |

| SOFTWISE | προσαρμογή εφαρμογής |  |  |  | 100,00 |
| --- | --- | --- | --- | --- | --- |
| SOFTWISE | Αξιοποίηση αποτελεσμάτων | Βιομηχανική Έρευνα (Άρθρο 25) | 45.817,75 € | 45.817,75 € | 100,00 |
| MEDPHYS | Απαιτήσεις χρηστών, αρχιτεκτονική συστήματος και προδιαγραφές | Βιομηχανική Έρευνα (Άρθρο 25) | 50.432,50 € | 50.432,50 € | 100,00 |
| MEDPHYS | Ενσωμάτωση απαιτήσεων, υλοποίηση και έλεγχος συστήματος | Βιομηχανική Έρευνα (Άρθρο 25) | 30.432,50 € | 30.432,50 € | 100,00 |
| MEDPHYS | Πιλοτικές δοκιμές, αξιολόγηση και προσαρμογή εφαρμογής | Πειραματική Ανάπτυξη (Άρθρο 25) | 57.932,50 € | 57.932,50 € | 100,00 |
| MEDPHYS | Αξιοποίηση αποτελεσμάτων | Βιομηχανική Έρευνα (Άρθρο 25) | 58.932,50 € | 58.932,50 € | 100,00 |

**4.2.2. ΚΑΤΑ ΤΗΝ ΑΞΙΟΛΟΓΗΣΗ**

| **ΦΟΡΕΑΣ** | **ΕΝΟΤΗΤΑ ΕΡΓΑΣΙΑΣ** | **ΚΑΤΗΓΟΡΙΑ ΔΡΑΣΤΗΡΙΟΤΗΤΑΣ** | **ΠΡΟΥΠΟΛΟΓΙΣΜΟΣ** | **ΔΗΜΟΣΙΑ ΔΑΠΑΝΗ** | **ΕΝΤΑΣΗ ΕΝΙΣΧΥΣΗΣ** |
| --- | --- | --- | --- | --- | --- |
| TSM | Απαιτήσεις χρηστών, αρχιτεκτονική συστήματος και προδιαγραφές | Βιομηχανική Έρευνα (Άρθρο 25) | 62.500,00 € | 50.000,00 € | 80,00 |
| TSM | Ενσωμάτωση απαιτήσεων, υλοποίηση και έλεγχος συστήματος | Βιομηχανική Έρευνα (Άρθρο 25) | 97.500,00 € | 78.000,00 € | 80,00 |
| TSM | Πιλοτικές δοκιμές, αξιολόγηση και προσαρμογή εφαρμογής | Πειραματική Ανάπτυξη (Άρθρο 25) | 45.000,00 € | 27.000,00 € | 60,00 |
| TSM | Αξιοποίηση αποτελεσμάτων | Βιομηχανική Έρευνα (Άρθρο 25) | 47.000,00 € | 37.600,00 € | 80,00 |
|  | Απαιτήσεις χρηστών, αρχιτεκτονική | Βιομηχανική Έρευνα (Άρθρο 25) | 49.187,75 € | 49.187,75 € |  |

| SOFTWISE | συστήματος και προδιαγραφές |  |  |  | 100,00 |
| --- | --- | --- | --- | --- | --- |
| SOFTWISE | Ενσωμάτωση απαιτήσεων, υλοποίηση και έλεγχος συστήματος | Βιομηχανική Έρευνα (Άρθρο 25) | 51.887,75 € | 51.887,75 € | 100,00 |
| SOFTWISE | Πιλοτικές δοκιμές, αξιολόγηση και προσαρμογή εφαρμογής | Πειραματική Ανάπτυξη (Άρθρο 25) | 68.087,75 € | 68.087,75 € | 100,00 |
| SOFTWISE | Αξιοποίηση αποτελεσμάτων | Βιομηχανική Έρευνα (Άρθρο 25) | 45.817,75 € | 45.817,75 € | 100,00 |
| MEDPHYS | Απαιτήσεις χρηστών, αρχιτεκτονική συστήματος και προδιαγραφές | Βιομηχανική Έρευνα (Άρθρο 25) | 50.432,50 € | 50.432,50 € | 100,00 |
| MEDPHYS | Ενσωμάτωση απαιτήσεων, υλοποίηση και έλεγχος συστήματος | Βιομηχανική Έρευνα (Άρθρο 25) | 30.432,50 € | 30.432,50 € | 100,00 |
| MEDPHYS | Πιλοτικές δοκιμές, αξιολόγηση και προσαρμογή εφαρμογής | Πειραματική Ανάπτυξη (Άρθρο 25) | 57.932,50 € | 57.932,50 € | 100,00 |
| MEDPHYS | Αξιοποίηση αποτελεσμάτων | Βιομηχανική Έρευνα (Άρθρο 25) | 58.932,50 € | 58.932,50 € | 100,00 |

**ΚΑΤΑΝΟΜΗ ΠΡΟΫΠΟΛΟΓΙΣΜΟΥ ΑΝΑ ΦΟΡΕΑ ΚΑΙ ΚΑΤΗΓΟΡΙΑ ΔΑΠΑΝΗΣ**

**4.3.**

| **4.3.1. ΚΑΤΑ ΤΗΝ ΥΠΟΒΟΛΗ** | |
| --- | --- |
| **Κατηγορία Δαπάνης** | **Εργαστήριο Τεχνολογίας Λογισμικού, Ιστού και Ευφυών Συστημάτων – ΤΜΗΜΑ Πληροφορικής-ΑΠΘ** |
| **Ενισχύσεις για έργα έρευνας και ανάπτυξης (Άρθρο 25)** | **214.981,00 €** |
| ΕΡ1 - Δαπάνες προσωπικού | 186.030,00 € |
| ΕΡ2 - Δαπάνες οργάνων και εξοπλισμού, κτιρίων και γηπέδων | 0,00 € |
| ΕΡ3 - Δαπάνες για έρευνα επί συμβάσει, γνώσεις και διπλώματα ευρεσιτεχνίας, συμβουλευτικές υπηρεσίες | 0,00 € |

| ΕΡ4 - Πρόσθετα γενικά έξοδα και λοιπές λειτουργικές δαπάνες | 5.000,00 € |
| --- | --- |
| ΜΕ1 - Δαπάνες για μελέτες τεχνικής σκοπιμότητας | 0,00 € |
| ΕΜΕΟ - Έμμεσες Λειτουργικές δαπάνες | 23.951,00 € |
| **Ενισχύσεις καινοτομίας για ΜΜΕ (Άρθρο 28)** | **0,00 €** |
| ΚΑ1 - Δαπάνες για ενισχύσεις καινοτομίας για ΜΜΕ | 0,00 € |
| **Ενισχύσεις για συμμετοχή ΜΜΕ σε εμπορικές εκθέσεις (Άρθρο 19)** | **0,00 €** |
| ΕΚ1 - Δαπάνες συμμετοχής ΜΜΕ σε εμπορικές εκθέσεις | 0,00 € |
| **ΣΥΝΟΛΟ** | **214.981,00 €** |

| **Κατηγορία Δαπάνης** | **ΕΡΓΑΣΤΗΡΙΟ ΙΑΤΡΙΚΗΣ ΦΥΣΙΚΗΣ – ΤΜΗΜΑ ΙΑΤΡΙΚΗΣ ΑΠΘ** |
| --- | --- |
| **Ενισχύσεις για έργα έρευνας και ανάπτυξης (Άρθρο 25)** | **197.730,00 €** |
| ΕΡ1 - Δαπάνες προσωπικού | 171.000,00 € |
| ΕΡ2 - Δαπάνες οργάνων και εξοπλισμού, κτιρίων και γηπέδων | 0,00 € |
| ΕΡ3 - Δαπάνες για έρευνα επί συμβάσει, γνώσεις και διπλώματα ευρεσιτεχνίας, συμβουλευτικές υπηρεσίες | 0,00 € |
| ΕΡ4 - Πρόσθετα γενικά έξοδα και λοιπές λειτουργικές δαπάνες | 5.000,00 € |
| ΜΕ1 - Δαπάνες για μελέτες τεχνικής σκοπιμότητας | 0,00 € |
| ΕΜΕΟ - Έμμεσες Λειτουργικές δαπάνες | 21.730,00 € |
| **Ενισχύσεις καινοτομίας για ΜΜΕ (Άρθρο 28)** | **0,00 €** |
| ΚΑ1 - Δαπάνες για ενισχύσεις καινοτομίας για ΜΜΕ | 0,00 € |
| **Ενισχύσεις για συμμετοχή ΜΜΕ σε εμπορικές εκθέσεις (Άρθρο 19)** | **0,00 €** |
| ΕΚ1 - Δαπάνες συμμετοχής ΜΜΕ σε εμπορικές εκθέσεις | 0,00 € |
| **ΣΥΝΟΛΟ** | **197.730,00 €** |

| **Κατηγορία Δαπάνης** | **THE SECOND METHOD ΑΝΩΝΥΜΗ ΕΤΑΙΡΕΙΑ ΚΑΙΝΟΤΟΜΩΝ ΥΠΗΡΕΣΙΩΝ ΕΡΕΥΝΣ ΚΑΙ ΑΝΑΠΤΥΞΗΣ ΕΦΑΡΜΟΓΩΝ ΥΠΟΛΟΓΙΣΤΗ ΚΑΙ ΟΠΤΙΚΟΑΚΟΥΣΤΙΚΗΣ ΠΑΡΑΓΩΓΗΣ** |
| --- | --- |
| **Ενισχύσεις για έργα έρευνας και ανάπτυξης (Άρθρο 25)** | **252.000,00 €** |
| ΕΡ1 - Δαπάνες προσωπικού | 220.000,00 € |
| ΕΡ2 - Δαπάνες οργάνων και εξοπλισμού, κτιρίων και γηπέδων | 0,00 € |
| ΕΡ3 - Δαπάνες για έρευνα επί συμβάσει, γνώσεις και διπλώματα ευρεσιτεχνίας, συμβουλευτικές υπηρεσίες | 4.000,00 € |
| ΕΡ4 - Πρόσθετα γενικά έξοδα και λοιπές λειτουργικές δαπάνες | 28.000,00 € |

| ΜΕ1 - Δαπάνες για μελέτες τεχνικής σκοπιμότητας | 0,00 € |
| --- | --- |
| ΕΜΕΟ - Έμμεσες Λειτουργικές δαπάνες | 0,00 € |
| **Ενισχύσεις καινοτομίας για ΜΜΕ (Άρθρο 28)** | **0,00 €** |
| ΚΑ1 - Δαπάνες για ενισχύσεις καινοτομίας για ΜΜΕ | 0,00 € |
| **Ενισχύσεις για συμμετοχή ΜΜΕ σε εμπορικές εκθέσεις (Άρθρο 19)** | **0,00 €** |
| ΕΚ1 - Δαπάνες συμμετοχής ΜΜΕ σε εμπορικές εκθέσεις | 0,00 € |
| **ΣΥΝΟΛΟ** | **252.000,00 €** |

| **Κατηγορία Δαπάνης** | **Σύνολο Επιλέξιμου Π/Υ** |
| --- | --- |
| **Ενισχύσεις για έργα έρευνας και ανάπτυξης (Άρθρο 25)** | **664.711,00 €** |
| ΕΡ1 - Δαπάνες προσωπικού | 577.030,00 € |
| ΕΡ2 - Δαπάνες οργάνων και εξοπλισμού, κτιρίων και γηπέδων | 0,00 € |
| ΕΡ3 - Δαπάνες για έρευνα επί συμβάσει, γνώσεις και διπλώματα ευρεσιτεχνίας, συμβουλευτικές υπηρεσίες | 4.000,00 € |
| ΕΡ4 - Πρόσθετα γενικά έξοδα και λοιπές λειτουργικές δαπάνες | 38.000,00 € |
| ΜΕ1 - Δαπάνες για μελέτες τεχνικής σκοπιμότητας | 0,00 € |
| ΕΜΕΟ - Έμμεσες Λειτουργικές δαπάνες | 45.681,00 € |
| **Ενισχύσεις καινοτομίας για ΜΜΕ (Άρθρο 28)** | **0,00 €** |
| ΚΑ1 - Δαπάνες για ενισχύσεις καινοτομίας για ΜΜΕ | 0,00 € |
| **Ενισχύσεις για συμμετοχή ΜΜΕ σε εμπορικές εκθέσεις (Άρθρο 19)** | **0,00 €** |
| ΕΚ1 - Δαπάνες συμμετοχής ΜΜΕ σε εμπορικές εκθέσεις | 0,00 € |
| **ΣΥΝΟΛΟ** | **664.711,00 €** |
| **4.3.2. ΚΑΤΑ ΤΗΝ ΑΞΙΟΛΟΓΗΣΗ** | |
| **Κατηγορία Δαπάνης** | **Εργαστήριο Τεχνολογίας Λογισμικού, Ιστού και Ευφυών Συστημάτων – ΤΜΗΜΑ Πληροφορικής-ΑΠΘ** |
| **Ενισχύσεις για έργα έρευνας και ανάπτυξης (Άρθρο 25)** | **214.981,00 €** |
| ΕΡ1 - Δαπάνες προσωπικού | 186.030,00 € |
| ΕΡ2 - Δαπάνες οργάνων και εξοπλισμού, κτιρίων και γηπέδων | 0,00 € |
| ΕΡ3 - Δαπάνες για έρευνα επί συμβάσει, γνώσεις και διπλώματα ευρεσιτεχνίας, συμβουλευτικές υπηρεσίες | 0,00 € |
| ΕΡ4 - Πρόσθετα γενικά έξοδα και λοιπές λειτουργικές δαπάνες | 5.000,00 € |
| ΜΕ1 - Δαπάνες για μελέτες τεχνικής σκοπιμότητας | 0,00 € |
| ΕΜΕΟ - Έμμεσες Λειτουργικές δαπάνες | 23.951,00 € |

| **Ενισχύσεις καινοτομίας για ΜΜΕ (Άρθρο 28)** | **0,00 €** |
| --- | --- |
| ΚΑ1 - Δαπάνες για ενισχύσεις καινοτομίας για ΜΜΕ | 0,00 € |
| **Ενισχύσεις για συμμετοχή ΜΜΕ σε εμπορικές εκθέσεις (Άρθρο 19)** | **0,00 €** |
| ΕΚ1 - Δαπάνες συμμετοχής ΜΜΕ σε εμπορικές εκθέσεις | 0,00 € |
| **ΣΥΝΟΛΟ** | **214.981,00 €** |

| **Κατηγορία Δαπάνης** | **ΕΡΓΑΣΤΗΡΙΟ ΙΑΤΡΙΚΗΣ ΦΥΣΙΚΗΣ – ΤΜΗΜΑ ΙΑΤΡΙΚΗΣ ΑΠΘ** |
| --- | --- |
| **Ενισχύσεις για έργα έρευνας και ανάπτυξης (Άρθρο 25)** | **197.730,00 €** |
| ΕΡ1 - Δαπάνες προσωπικού | 171.000,00 € |
| ΕΡ2 - Δαπάνες οργάνων και εξοπλισμού, κτιρίων και γηπέδων | 0,00 € |
| ΕΡ3 - Δαπάνες για έρευνα επί συμβάσει, γνώσεις και διπλώματα ευρεσιτεχνίας, συμβουλευτικές υπηρεσίες | 0,00 € |
| ΕΡ4 - Πρόσθετα γενικά έξοδα και λοιπές λειτουργικές δαπάνες | 5.000,00 € |
| ΜΕ1 - Δαπάνες για μελέτες τεχνικής σκοπιμότητας | 0,00 € |
| ΕΜΕΟ - Έμμεσες Λειτουργικές δαπάνες | 21.730,00 € |
| **Ενισχύσεις καινοτομίας για ΜΜΕ (Άρθρο 28)** | **0,00 €** |
| ΚΑ1 - Δαπάνες για ενισχύσεις καινοτομίας για ΜΜΕ | 0,00 € |
| **Ενισχύσεις για συμμετοχή ΜΜΕ σε εμπορικές εκθέσεις (Άρθρο 19)** | **0,00 €** |
| ΕΚ1 - Δαπάνες συμμετοχής ΜΜΕ σε εμπορικές εκθέσεις | 0,00 € |
| **ΣΥΝΟΛΟ** | **197.730,00 €** |

| **Κατηγορία Δαπάνης** | **THE SECOND METHOD ΑΝΩΝΥΜΗ ΕΤΑΙΡΕΙΑ ΚΑΙΝΟΤΟΜΩΝ ΥΠΗΡΕΣΙΩΝ ΕΡΕΥΝΣ ΚΑΙ ΑΝΑΠΤΥΞΗΣ ΕΦΑΡΜΟΓΩΝ ΥΠΟΛΟΓΙΣΤΗ ΚΑΙ ΟΠΤΙΚΟΑΚΟΥΣΤΙΚΗΣ ΠΑΡΑΓΩΓΗΣ** |
| --- | --- |
| **Ενισχύσεις για έργα έρευνας και ανάπτυξης (Άρθρο 25)** | **252.000,00 €** |
| ΕΡ1 - Δαπάνες προσωπικού | 220.000,00 € |
| ΕΡ2 - Δαπάνες οργάνων και εξοπλισμού, κτιρίων και γηπέδων | 0,00 € |
| ΕΡ3 - Δαπάνες για έρευνα επί συμβάσει, γνώσεις και διπλώματα ευρεσιτεχνίας, συμβουλευτικές υπηρεσίες | 4.000,00 € |
| ΕΡ4 - Πρόσθετα γενικά έξοδα και λοιπές λειτουργικές δαπάνες | 28.000,00 € |
| ΜΕ1 - Δαπάνες για μελέτες τεχνικής σκοπιμότητας | 0,00 € |
| ΕΜΕΟ - Έμμεσες Λειτουργικές δαπάνες | 0,00 € |

| **Ενισχύσεις καινοτομίας για ΜΜΕ (Άρθρο 28)** | **0,00 €** |
| --- | --- |
| ΚΑ1 - Δαπάνες για ενισχύσεις καινοτομίας για ΜΜΕ | 0,00 € |
| **Ενισχύσεις για συμμετοχή ΜΜΕ σε εμπορικές εκθέσεις (Άρθρο 19)** | **0,00 €** |
| ΕΚ1 - Δαπάνες συμμετοχής ΜΜΕ σε εμπορικές εκθέσεις | 0,00 € |
| **ΣΥΝΟΛΟ** | **252.000,00 €** |

| **Κατηγορία Δαπάνης** | **Σύνολο Επιλέξιμου Π/Υ** |
| --- | --- |
| **Ενισχύσεις για έργα έρευνας και ανάπτυξης (Άρθρο 25)** | **664.711,00 €** |
| ΕΡ1 - Δαπάνες προσωπικού | 577.030,00 € |
| ΕΡ2 - Δαπάνες οργάνων και εξοπλισμού, κτιρίων και γηπέδων | 0,00 € |
| ΕΡ3 - Δαπάνες για έρευνα επί συμβάσει, γνώσεις και διπλώματα ευρεσιτεχνίας, συμβουλευτικές υπηρεσίες | 4.000,00 € |
| ΕΡ4 - Πρόσθετα γενικά έξοδα και λοιπές λειτουργικές δαπάνες | 38.000,00 € |
| ΜΕ1 - Δαπάνες για μελέτες τεχνικής σκοπιμότητας | 0,00 € |
| ΕΜΕΟ - Έμμεσες Λειτουργικές δαπάνες | 45.681,00 € |
| **Ενισχύσεις καινοτομίας για ΜΜΕ (Άρθρο 28)** | **0,00 €** |
| ΚΑ1 - Δαπάνες για ενισχύσεις καινοτομίας για ΜΜΕ | 0,00 € |
| **Ενισχύσεις για συμμετοχή ΜΜΕ σε εμπορικές εκθέσεις (Άρθρο 19)** | **0,00 €** |
| ΕΚ1 - Δαπάνες συμμετοχής ΜΜΕ σε εμπορικές εκθέσεις | 0,00 € |
| **ΣΥΝΟΛΟ** | **664.711,00 €** |

| **4.4.** | **ΚΑΤΑΝΟΜΗ ΠΡΟΫΠΟΛΟΓΙΣΜΟΥ ΚΑΙ ΔΗΜΟΣΙΑΣ ΔΑΠΑΝΗΣ** | | | | | | | |
| --- | --- | --- | --- | --- | --- | --- | --- | --- |
| **4.4.1. ΚΑΤΑ ΤΗΝ ΥΠΟΒΟΛΗ** | | | | | | | | |
| **Α/Α ΦΟΡΕΑ** | | **ΣΥΝΤΟΜΟΓΡ ΑΦΙΑ ΦΟΡΕΑ** | **ΕΙΔΟΣ ΦΟΡΕΑ** | **ΠΡΟΫΠ/ΣΜΟΣ** | **ΔΗΜΟΣΙΑ ΔΑΠΑΝΗ** | **ΔΗΜΟΣΙΑ ΔΑΠΑΝΗ (%)** | **(%) Δ.Δ. ΕΠΙ ΤΗΣ ΣΥΝΟΛΙΚΗΣ Δ.Δ.** | **ΠΕΡΙΦΕΡΕΙΕΣ** |
| 1 | | SOFTWISE | Ερευνητικός Οργανισμός | 214.981,00 € | 214.981,00 € | 100,00 | 35,52 | Κεντρικής  Μακεδονίας |
| 2 | | MEDPHYS | Ερευνητικός Οργανισμός | 197.730,00 € | 197.730,00 € | 100,00 | 32,67 | Κεντρικής  Μακεδονίας |
| 3 | | TSM | Επιχείρηση | 252.000,00 € | 192.600,00 € | 76,43 | 31,82 | Κεντρικής |

|  |  |  |  |  |  |  | Μακεδονίας |
| --- | --- | --- | --- | --- | --- | --- | --- |
| **ΣΥΝΟΛΟ** | | | 664.711,00 € | 605.311,00 € | 91,06 | 100,00 |  |
| **4.4.2. ΚΑΤΑ ΤΗΝ ΑΞΙΟΛΟΓΗΣΗ** | | | | | | | |
| **Α/Α ΦΟΡΕΑ** | **ΣΥΝΤΟΜΟΓΡ ΑΦΙΑ ΦΟΡΕΑ** | **ΕΙΔΟΣ ΦΟΡΕΑ** | **ΠΡΟΫΠ/ΣΜΟΣ** | **ΔΗΜΟΣΙΑ ΔΑΠΑΝΗ** | **ΔΗΜΟΣΙΑ ΔΑΠΑΝΗ (%)** | **(%) Δ.Δ. ΕΠΙ ΤΗΣ ΣΥΝΟΛΙΚΗΣ Δ.Δ.** | **ΠΕΡΙΦΕΡΕΙΕΣ** |
| 1 | SOFTWISE | Ερευνητικός Οργανισμός | 214.981,00 € | 214.981,00 € | 100,00 | 35,52 | Κεντρικής  Μακεδονίας |
| 2 | MEDPHYS | Ερευνητικός Οργανισμός | 197.730,00 € | 197.730,00 € | 100,00 | 32,67 | Κεντρικής  Μακεδονίας |
| 3 | TSM | Επιχείρηση | 252.000,00 € | 192.600,00 € | 76,43 | 31,82 | Κεντρικής  Μακεδονίας |
| **ΣΥΝΟΛΟ** | | | 664.711,00 € | 605.311,00 € | 91,06 | 100,00 |  |

*ΕΝΤΥΠΟ ΑΞΙΟΛΟΓΗΣΗΣ ΠΡΟΤΑΣΗΣ ΕΡΕΥΝΗΤΙΚΟΥ ΕΡΓΟΥ*

**ΣΥΝΟΛΙΚΗ ΒΑΘΜΟΛΟΓΙΑ**

**5.**

| **Κριτήριο** | **Βαθμολογία επιμέρους κριτηρίου** | **Συντελε στής βαρύτη τας (%)** | **Τελική Βαθμολογία Κριτηρίου** |
| --- | --- | --- | --- |
| **Α Επιστημονική και τεχνική αρτιότητα του**  **προτεινόμενου έργου (Excellence) (Παρέμβαση ΙΙ)** | 3,00 | 40,00 | 1,20 |
| **Β Εμπειρία και αξιοπιστία του (ων) δικαιούχου (ων) και ποιότητα και ικανότητα του τρόπου υλοποίησης του έργου (Implementation)**  **(Παρέμβαση ΙΙ)** | 3,00 | 30,00 | 0,90 |
| **Γ Αποτελέσματα και Επιπτώσεις του προτεινόμενου σχεδίου (Impact) (Παρέμβαση ΙΙ)** | 3,00 | 30,00 | 0,90 |
| **Συνολική Βαθμολογία** | | | **3,00** |
| **Ικανοποίηση Βαθμολογικού Κριτηρίου** | | | **ΝΑΙ** |

**ΑΠΟΤΕΛΕΣΜΑ ΑΞΙΟΛΟΓΗΣΗΣ**

**6.**

Εγκριθείσα

**Τελικό Αποτέλεσμα Αξιολόγησης:**
